# Direct notification by health professionals of relatives at-risk of genetic conditions (with patient consent): views of the Australian public

**DOI:** 10.1038/s41431-023-01395-9

**Published:** 2023-06-06

**Authors:** Jane M. Tiller, Ami Stott, Keri Finlay, Tiffany Boughtwood, Evanthia O. Madelli, Ari Horton, Ingrid Winship, Kristen Nowak, Margaret Otlowski

**Affiliations:** 1 Australian Genomics, Parkville, VIC Australia; 2 Murdoch Children’s Research Institute, Parkville, VIC Australia; 3 Department of Epidemiology and Preventive Medicine, Monash University, Melbourne, VIC Australia; 4 Department of Paediatrics, Monash University, Clayton, VIC Australia; 5 Department of Genomic Medicine, The Royal Melbourne Hospital, Parkville, VIC Australia; 6 Office of Population Health Genomics, WA Department of Health, East Perth, WA Australia; 7 Centre for Law and Genetics, University of Tasmania, Hobart, TAS Australia

**Keywords:** Genetics, Health care, Clinical genetics, Ethics

## Abstract

Genetic risk information for medically actionable conditions has relevance for patients’ blood relatives. However, cascade testing uptake in at-risk families is <50%, and the burden of contacting relatives is a significant barrier to dissemination of risk information. Health professionals (HPs) could notify at-risk relatives directly, with patients’ consent. This practice is supported by international literature, including strong public support. However, there is little exploration of the Australian public’s views about this issue. We surveyed Australian adults using a consumer research company. Respondents were provided a hypothetical scenario and asked about views and preferences regarding direct contact by HPs. 1030 members of the public responded, with median age 45 y and 51% female. The majority would want to be told about genetic risk for conditions that can be prevented/treated early (85%) and contacted directly by a HP (68%). Most preferred a letter that included specific information about the genetic condition in the family (67%) and had no privacy concerns about HPs sending a letter using contact details provided by a relative (85%). A minority (< 5%) had significant privacy concerns, mostly about use of personal contact information. Concerns included ensuring information was not shared with third parties. Almost 50% would prefer that a family member contacted them before the letter was sent, while about half did not prefer this or were unsure. The Australian public supports (and prefers) direct notification of relatives at risk of medically actionable genetic conditions. Guidelines would assist with clarifying clinicians’ discretion in this area.

## Introduction

Genetic risk information for medically actionable conditions has the potential to save lives, through prevention and/or early detection and treatment of disease. Such genetic information is familial in nature, meaning that the information is shared across families, and individual genetic information has potential implications for family members related by blood [1].

For patients with genetic test results that indicate an increased risk of disease, post-test genetic counselling should involve a discussion of the implications for their own health, as well as for their blood relatives [2]. Some health professionals (HPs) provide “family letters” for patients to pass to relatives, with information about their possible genetic risk and availability of testing. However, many HPs fail to provide this assistance [3] and many family members do not pass such information to their at-risk relatives [4, 5].

“Cascade testing” (subsequent testing of relatives after the first individual is identified) is one of the most significant methods for efficient, cost-effective identification of at-risk individuals in the community. This is most beneficial before disease onset, while prevention or early intervention is most effective [6–8]. Nevertheless, uptake of cascade testing in at-risk families is poor, and numerous Australian and international publications have identified the need to improve the uptake of cascade testing [5, 7–11]. A recent systematic review and meta-analysis of 87 international studies found that less than 50% of relatives underwent cascade genetic counselling and/or genetic testing [12]. Family communication is often fraught, and many at-risk individuals remain unaware of their eligibility for genetic testing and preventive measures. The public health implications of failing to notify at-risk relatives, so potentially life-saving prevention or early treatment that can be realised, make family risk notification a public health priority [5, 7–11].

A recent international review found that family dynamics, communication concerns, difficult disclosure experiences and emotional distance are barriers for both disclosure to relatives and uptake of testing by relatives [5]. In over half of families in a 2017 Australian study (*n* = 165) at least one at-risk relative had not been notified of their potential genetic risk [4]. The responsibility of contacting relatives was a significant barrier to dissemination, and affected (symptomatic) individuals more commonly left contact to other relatives, demonstrating the increased barrier of contact responsibility for symptomatic individuals. One method to increase dissemination of risk information to relatives is direct contact by HPs with patient consent, where patients provide HPs with contact details of their at-risk relatives and consent to the HP notifying them of their genetic risk and testing options. Increasingly, relevant HPs may include clinical geneticists and genetic counsellors, as well as oncologists, nurses, and other HPs who assist patients with contacting relatives after genetic testing.

International literature supports the effectiveness of this approach, including strong public and patient support [7, 11, 13–19], with many studies recommending that HPs consider contacting at-risk patients directly. A 2016 Belgian study found that HPs directly informing relatives almost doubled the number of relatives tested for *BRCA1/2* mutations, and that it was psychologically safe [14]. A 2009 UK study also demonstrated increased uptake of *BRCA1/2* cascade testing where relatives were contacted directly by HPs [20]. A 2007 Finnish study in families with Lynch Syndrome found strong acceptability and good psychosocial outcomes for relatives contacted directly by HPs [13]. In the Netherlands, direct contact of relatives of familial hypercholesterolaemia (FH) patients was part of a screening program introduced in 1994, which identified 28,000 FH patients between 1994–2014 [21]. When funding was reduced in 2014 and direct contact methods ceased, the number of family members identified dropped from 2000/year to ~400 in 2015 [22], leading to calls from HPs to urgently reinstate the successful program [23]. This was not done, and a 2020 Dutch study found that both patients and the general population felt that HPs should be actively involved in informing at-risk relatives [11]. France has perhaps the strongest known health policy supporting dissemination of genetic risk information–patients have a legal obligation to disclose relevant information to their relatives at-risk of a genetic condition, and can choose to disclose the information personally or to allow a HP to notify the relatives directly [24]. A US-based review found that direct notification by HPs increased cascade testing uptake [7], and a 2021 US study showed direct contact of at-risk relatives of patients with hereditary cancer syndromes was acceptable and should be incorporated into practice [18]. A 2022 systematic review and meta-analysis of 87 international studies found direct contact increased uptake of cascade genetic testing from 40% to 62% [12].

In Australia, in 2006 the South Australian Clinical Genetics Service compared cascade testing uptake where patients were i) given a letter to share with at-risk relatives, or ii) asked for consent to send letters directly to their at-risk relatives [25]. The study found that direct notification by HPs almost doubled cascade testing uptake, and no relatives who were contacted directly raised breach of privacy or other concerns (echoed by a Finnish study, where negative reactions were anticipated but did not occur [13]). Historical studies in Australia which have focussed on cascade testing for FH have demonstrated that both general consumers [26] and patients [27] are strongly supportive of direct contact by HPs. Published FH guidelines detail considerations for direct risk-notification by HPs [28]. However, clinicians anecdotally continue to express uncertainty about this practice, and little is known about the Australian public’s current views about being contacted in this way.

## Methods

### Survey design

We developed an online survey, designed to gather the general public’s views regarding direct risk notification by HPs, preferences about contact methods and concerns about receiving genetic information this way. Surveys were initially designed by a team of researchers including clinicians, academics, legal and policy experts, and public health personnel. Prior to full launch, it was piloted on 100 respondents, and the data reviewed to ensure consumer understanding.

Initially, respondents were introduced to the concept of medically actionable genetic conditions, and the importance of sharing information with at-risk family members (see Box 1).

Respondents were then provided with copies of 2 example letters (Supplementary Files S1 and S2) and asked questions about their views and preferences. Letter S1 contains general information about a genetic condition in the family that may affect the recipient’s health. Letter S2 contains more specific information about the family variant, the associated health risks, and preventive measures that are available.

Box 1 Introductory information provided to survey respondents Genetic testing for certain DNA variants can tell us about high risks of developing health conditions in the future, like some cancers or cardiac conditions. Often these are conditions that can be prevented, or detected and treated early.

Because DNA is inherited (passed from parents to children), this risk can run in families. When one family member finds out about this type of risk, their managing health professional will usually advise them to tell their blood relatives about the possibility that they might also have the same DNA variants and the same health risks.

In this survey, we will ask for your thoughts about receiving this type of genetic information from a health professional. Don’t worry if you don’t know too much about this topic – we are interested in your views, no matter your level of knowledge.

### Recruitment

Consumers were eligible if they were ≥18 years old and living in Australia. We distributed the survey (Supplementary File S3) through Dynata, an internationally recognised consumer research company [29]. Dynata follows local privacy and data protection laws, and maintains Australian certification with the Research Society Fair Data Accreditation (certificate number ISOEX-110011-2). Dynata has a large consumer database of potential survey participants who voluntarily sign up to be offered surveys to complete. They are provided with points on successful completion of surveys, which can be used to redeem rewards. Dynata was contracted to obtain at least 1000 completed responses, with an even spread across age, sex, and geographic location. Given its large consumer database, Dynata are able to access a representative sample of Australian adults.

### Analysis

Data were analysed using STATA 17 statistical analysis software [30]. Descriptive statistical analysis was used to summarise the categorical variables including the self-reported demographic information collected. Cross tabulation analysis was used to identify the relationship between multiple variables, and Pearson’s chi-square tests were conducted to establish significance for any differences. A record of the STATA analysis and chi-square tests is included as a pdf of the do file at Supplementary File S4.

Free text comments were grouped into themes, to provide insight, explanation and richer context for the survey responses.

## Results

Overall, 1030 members of the public responded over a period of 3 days. Because recruitment was ceased soon after reaching 1000 respondents, we are unable to determine a response rate. We do not know how many consumers in the Dynata database viewed the research opportunity in order to obtain 1030 responses. Table 1 shows self-reported demographic characteristics of respondents, as well as demographic breakdown of the Australian population, based on the 2021 Australian Bureau of Statistics census [31]. The demographic characteristics of the surveyed population are very similar to the Australian population data, indicating a strong representative sample across sex, age, geographical location, and level of education. The median age of our cohort was 45 years and 50% were female.

**Table 1:** Respondents’ demographic characteristics, with comparative Australian population demographics.

*n* = 1030		*n*	%	Australian population (%)^a^
Sex	Male	502	49.2	49.3
	Female	514	50.4	50.7
	Other	4	0.4	0.17
Age range (years)	18–24	100	9.9	11.2
	25–40	276	27.4	29.8
	41–60	376	37.3	31.8
	61–80	241	23.9	22.5
	>81	14	1.4	4.8
State/ Territory of residence	Australian Capital Territory	22	2.2	1.8
	New South Wales	325	32.5	31.8
	Northern Territory	2	0.2	0.9
	Queensland	201	20.1	20.3
	South Australia	81	8.1	7
	Tasmania	20	2	2.2
	Victoria	254	25.4	25.6
	Western Australia	95	9.5	10.5
Location (self-reported**)**	Rural	263	26.2	28.3
	Urban	742	73.8	71.7
Highest level of education completed	<Yr12	135	13.2	20.9
	Year 12	186	18.2	17.9
	TAFE/diploma	327	31.9	33.3
	Undergraduate degree	247	24.1	17.7
	Post-graduate degree	129	12.6	8.1

^a^Source: Australian Bureau of Statistics 2021 Census: www.abs.gov.au/statistics/people/population/population-census.

### Preferences regarding contact

A large majority (85%) of respondents would want to be told by a HP about genetic risk for future health problems that can be prevented or detected/treated early (Table 2). Of those, 43% (*n* = 376/872) initially said they would prefer to be told the information up front, whereas 57% said they would prefer to be told the information exists and be given the option to find out more.

**Table 2:** Preferences regarding being contacted directly by health professionals about medically actionable genetic risk information.

*n* = 1030		*n*	%
What are your thoughts about being told (by a health professional) about genetic information that might show that you are at risk for future health problems that can be prevented (or detected and treated early)?	I would want to be contacted and told that this information exists, and be given the option to find out more or not	496	48.2
	I would want to be contacted and told up-front what this information is and what the health risks to me are	376	36.5
	I would not want to be contacted to be offered this type of genetic information	158	15.3
Thoughts about Letter 1, which did not say which condition the hypothetical family member’s test relates to	I would have felt better informed if the genetic condition had been included in the letter	749	72.7
	Including the genetic condition in the letter would have been overwhelming for me at this early stage	250	24.3
	Other	31	3.0
Thoughts about Letter 2, which contains more information about the genetic condition	I felt better informed from this letter than from the previous letter	775	75.2
	It felt overwhelming to have that level of information at this early stage	231	22.4
	Other	24	2.3
Which letter do you prefer?	Letter 1 (no specific information about the genetic condition)	217	21.1
	Letter 2 (specific information about the genetic condition)	691	67.1
	No preference	122	11.8
How easy was letter 1 to understand?	Very easy to understand	428	41.6
	Easy enough to understand	497	48.3
	A bit difficult to understand	89	8.6
	Very difficult to understand	16	1.6
How easy was letter 2 to understand?	Very easy to understand	454	44.1
	Easy enough to understand	473	45.9
	A bit difficult to understand	91	8.8
	Very difficult to understand	12	1.2
Would you have any privacy concerns about being sent a letter directly by a health professional, using details provided to them by your relative?	No concerns	878	85.2
	A little concern	104	10.1
	Significant concerns	48	4.7
Would you have any other (non-privacy related) concerns about being sent a letter directly in this way?	No concerns	946	91.8
	A little concern	57	5.5
	Significant concerns	27	2.6

After reviewing Letter 1 (S1), which did not contain specific genetic information, 73% said they would have felt better informed if the genetic condition had been included in the letter, whereas 24% thought that would have been overwhelming at this early stage. After reviewing with Letter 2 (S2), which does contain specific genetic information, 76% felt better informed by this letter, whereas 22% said it felt overwhelming. There was some movement of preferences in both directions, though generally more towards preferring more genetic information. Of respondents who said they felt including the genetic condition would be overwhelming after reading Letter 1, 41% (*n* = 103/250) felt better informed after reading Letter 2. Conversely, of respondents who said they would have felt better knowing the genetic condition after reading Letter 1, only 11% (*n* = 83/749) said it felt overwhelming after reading Letter 2.

Overall, the majority (67%) of respondents preferred the letter with more genetic information (S2), while only 21% preferred the letter without specific genetic information (S1); 12% had no preference (Table 2). This preference for letter S2 became more pronounced with increasing age [χ^2^ = 17.46, *P* < 0.05]. No significant differences were observed in preferences associated with sex, location, or level of education. The majority of respondents (90%) rated both letters as very easy/easy enough to understand, with <2% choosing “very difficult to understand” for each letter.

In free text comments (*n* = 197) about the two letters (Table 3), those who preferred Letter 1 primarily stated that the initial letter should not contain too much information (34%; *n* = 12/35); Letter 1 was easier to understand (26%; *n* = 9/35); and had some suspicions about the source/whether the letter was a scam (17%; *n* = 6/35). Several respondents who preferred Letter 1 also noted that both letters were acceptable (9%; *n* = 3/35) and they would be grateful for the opportunity to learn about the information (6%; *n* = 2/35).

**Table 3:** Categories of free-text comments regarding preference for Letter 1 or Letter 2.

		Category	*n* (%)	Example quote/s
Which letter would you prefer? [free text]	Letter 1 (*n* = 35)	The initial letter should not have too much detailed information/the right not to know	12 (34.3)	*“I do like that the first letter holds back the information unless you request it. Receiving a letter like the second could create anxiety and panic amongst some people. And they’d misinterpret the meaning of the letter.” (ID 770, M, 37* *y)*
				*“I think some people want to decide for themselves if they want to find out about possible genetic conditions and should be able to choose if they find out or not” (ID 430, F, 31* *y)*
		Easier to understand	9 (25.7)	*“Letter one is easier to understand and felt warmer” (ID 317, F, 28* *y)*
		Suspicion about source	6 (17.1)	*“With the way Letter 2 started off, ‘I am a medical specialist…’ felt very fake. I would feel suspicious” (ID, 288, F, 22* *y)*
		More information required	3 (8.6)	*“I think maybe a statement if it was an urgent request or the level of seriousness to take this letter with so you know if it’s an urgent matter and don’t panic if it’s not necessary.” (ID 408, F, 33* *y)*
		Both letters acceptable	3 (8.6)	*“Both are great, nothing need [sic] to change” (ID 955, F, 40* *y)*
		Positive/grateful for the opportunity	2 (5.7)	*“I liked how they at least notified me about such a problem - it’s better to know of this than to not know at all.” (ID 626, M, 41* *y)*
	Letter 2 (*n* = 144)	This letter was more informative/impresses the urgency of situation	74 (51.4)	*“After reading the first letter I thought I would feel overwhelmed by more information, however the 2nd letter had just the right amount of information to not feel overwhelmed, but know what may happen and what we could do about it.” (ID 618, M, 43* *y)*
				*“Definitely would prefer to receive the 2nd letter as this would impress on me the immediacy of the situation therefore potentially saving my life or the very least buy me more time” (ID 548, F, 63* *y)*
		Both letters acceptable	19 (13.2)	*“I feel that the penmanship of both letters is of an acceptable standard and no issues understanding it. But letter no.2 definitely is the superior version.” (ID 575, M, 40* *y)*
		More information required about the condition/ relative	15 (10.4)	*“I found that they should’ve put more information about the genetic condition as some people aren’t tech savvy and might not be able to research the certain disease” (ID 374, F, 22* *y)*
				*“In the letter it needs to state the relative’s name, otherwise it would be taken as a scam.” (ID 1059, F, 51* *y)*
		Positive/grateful for the opportunity	13 (9.0)	*“I really liked it. I think that if the family member didn’t have my contact details, I would be disappointed that it could go missed.” (ID 177, F, 30* *y)*
		Letter was easy to follow	9 (6.3)	*“It was communicated so that anyone can understand it” (ID 951, F, 36* *y)*
		Prefer not to be contacted this way/would want to hear from the relative	7 (4.9)	*“I would have preferred to have not received any letter at all. If I was to be informed, I would prefer to have a face to face conversation with whoever has the information…. I prefer not to know about maybe’s. I would deal with situation when it or if it arises.” (ID 801, M, 71* *y)*
				*“I would have expected my relative to advise me about their situation and expect to be contacted by letter later on” (ID 698, M, 70* *y)*
		Suspicion about source	5 (3.5)	*“I would want to know who the specialist is & their qualifications eg Prof Jones PHD, MD etc. It reads like a chain letter saying the person is a specialist and may not be taken seriously.” (ID 945, F, 61* *y)*
		Tone could be softened	2 (1.4)	*“The first letter was too vague and the second was considerably better although the second letter would be improved if it had a more sympathetic tone” (ID 144, F, 20* *y)*
	No preference (*n* = 18)	Both letters acceptable	5 (27.8)	*“I thought they were both great and I would be happy to receive either” (ID 1051, F, 36* *y)*
		Privacy concerns	5 (27.8)	*“I would be very uncomfortable receiving such a letter and I would complain to the people who send it in the first place as well as tell my relative off for passing on my information. Both intrusive parties here have no right to do either.” (ID 1022, F, 53* *y)*
		Prefer not to be contacted this way/would want to hear from the relative	4 (22.2)	*“Would not like to get this letter then get anxious thinking about something that may not even happen” (ID 156, F, 32* *y)*
				*“I would prefer to be told directly from the relative rather that the first indication being a letter received out of the blue.” (ID 625, M, 53* *y)*
		Suspicion about source	3 (16.7)	*“I just can’t imagine I would take it seriously if it really did arrive out of the blue.” (ID 164, F, 27* *y)*
		More information required	1 (5.6)	*“Including the name who was tested for the gene would have helped.” (ID 418, F, 22* *y)*

The majority of those who preferred Letter 2 stated it was more informative and/or impressed the urgency of the situation better than Letter 1 (51%; *n* = 74/144); felt both letters were acceptable (13%; n = 19/144); and preferred even more information about the condition/the relative be provided (10%; *n* = 15/144). Although some provided positive comments about the opportunity (9%; *n* = 13/144), others stated they would prefer not to be contacted this way (5%; *n* = 7/144) or that they were suspicious of the source (4%; *n* = 5/144). A small number of comments from those who stated they had no preference indicated these respondents felt both letters were acceptable (28%; *n* = 5/18); or that they had privacy concerns (28%; *n* = 5/18), preferred not to be contacted this way (22%; *n* = 4/18) or were suspicious of the source of the letter (17%; *n* = 3/18).

**Fig. 1:**
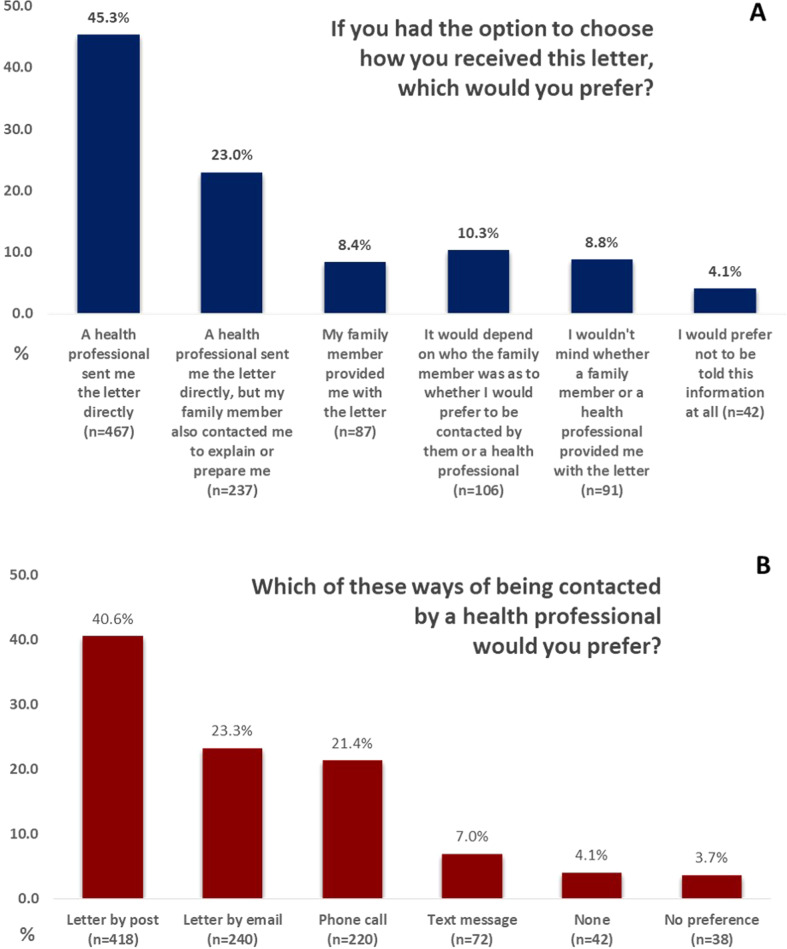
Respondent preferences regarding methods of notification about genetic risk.

Regarding preferences for receiving the letter (Fig. 1A), 68% would prefer a HP sent them the letter directly-a portion of those also preferred that a family member contacted them first (34%; *n* = 237/704). Only 8% preferred their family member provided them with the letter, although an additional 10% said it would depend on who the family member was, and 9% wouldn’t mind either way. In this question, only 4% would prefer not to be told about this information at all.

When asked about ways of providing this information (multiple options could be selected), the majority (62%) chose a letter by post, with letter by email (37%) and phone call (36%) the next most common. A text message (15%) was the least chosen option. When asked for a preference (Fig. 1B), letter by post (41%) was most preferred; text message (7%) was least preferred. These preferences remained across all age groups. Although younger people (18–40 y; 13%; *n* = 48/376); seemed more likely to prefer a text message than older people (> 40 y; 3%;*n* = 22/631), it was still the least preferred method of contact for younger age groups. There were no differences in preferences between respondents in self-reported rural and urban areas.

Few respondents (< 50) provided optional free text comments about preferences for methods of receiving this information. However, of those who preferred to be sent a letter directly by a HP, most commented about the credibility of medical source (50%; *n* = 6/12) or that family dynamics may make it difficult to get a letter from relatives (33%; *n* = 4/12). Those who wanted a relative to contact them in addition to the HP mentioned this would increase credibility (50%; *n* = 6/12) and allow them prepare (33%; *n* = 4/12). Of those who preferred only to be told by a relative, 33% (*n* = 2/6) would think it was a ‘scam’ if it didn’t come directly from relatives, and 50% (*n* = 3/6) considered this information should come from family first.

When asked specifically, 46% of respondents would prefer a relative contact them first before being contacted directly; 32% said no and 22% were unsure. Younger (18–24 y) and older ( > 81 y) age groups preferred contact by a relative in greater proportions than the age groups in between [χ^2^ = 18.76, *P* < 0.05]. Females also showed a slightly higher preference than males for contact by family members before HP contact [χ^2^ = 15.79, *P* < 0.05]. Level of education was not associated with any differences in preference.

### Privacy and non-privacy concerns

Respondents were asked whether they would have privacy concerns or other (non-privacy) concerns about being sent a letter directly by a HP, using details provided by their relative (Table 2). A large majority of respondents had no privacy concerns (85%) or non-privacy concerns (92%). A minority had significant privacy concerns (5%) and non-privacy concerns (3%). Of 1030 respondents, 83% had no concerns (privacy or non-privacy) at all. Of those who had significant non-privacy concerns, 89% also had significant privacy concerns (*n* = 24/27).

Although the numbers are small, a higher level of education seemed to show an association with higher privacy concerns [χ^2^ = 22.85, *P* < 0.05]. Of those with significant privacy concerns, 63% (*n* = 30/48) were undergraduates/post-graduates, who made up only 37% of all respondents (*n* = 376/1024). Non-privacy concerns seemed to follow the same trend, but with even smaller numbers, the significance is difficult to assess. Similarly, respondents’ (self-reported) living region seemed to show an association with privacy concerns - those from urban areas had more privacy concerns than those from rural areas [χ^2^ = 11.02, *P* < 0.05].

Respondents with significant concerns or a little concern could elaborate in free text (Table 4). Of 127 respondents who provided free-text comments regarding privacy concerns, 33% of those with significant concerns (*n* = 13/40) and 25% of those with a little concern (*n* = 22/87) raised the fact that their contact details had been provided to the HP without their prior permission. Concerns about how their personal information might be used, or accessed by other entities such as insurance companies, were raised by 25% (*n* = 10/40) and 13% (*n* = 11/87) of those with significant/little concerns respectively. Other concerns included the possibility that the letter could be a scam, general privacy concerns, worries that letters could go missing or be opened by others, and preferences regarding prior contact from relatives. Some made comments about the emotional impact of such information, which is arguably a non-privacy concern. Of 60 respondents who provided free-text comments regarding non-privacy concerns, 24% (*n* = 5/21) and 26% (*n* = 10/39) respectively of those with significant/little concerns also commented on the emotional impact of the information. Other matters listed as non-privacy concerns tended to overlap with listed privacy concerns.

**Table 4:** Categories of free text responses regarding privacy and non-privacy concerns associated with direct notification by health professionals.

		Main categories	*n* (%)	Example quote/s
PRIVACY CONCERNS (*n* = 127)	A little concern (*n* = 87)	Use/provision of contact details without permission	22 (25.3)	*“My relative has no right to provide my contact details to a doctor” (ID 520, F, 44* *y)*
				*“I would prefer to have been asked my permission first” (ID 911, M, 44* *y)*
		Use of personal information	11 (12.6)	*“How is this genetic linking information stored, shared and managed by the health professional” (ID 647, M, 41* *y)*
				*“This information being sent to insurance company” (ID 75, F, 33* *y)*
		Possibility of letter being a scam/marketing	11 (12.6)	*“My only concern is whether it was genuine. In other words, given all the scams which take place these days over a range of topics, how do I know it isn’t a scam of some type.” (ID 511, M, 57* *y)*
		Reliability of delivery method	10 (11.5)	*“Seems like an archaic form of communication for something so serious” (ID 136, F, 25* *y)*
				*“The only concern I would have is if the letter was opened by someone else” (ID 338, M, 28* *y)*
		Privacy/confidentiality concern	8 (9.2)	*“All medical information about you should always be confidential” (ID 635, M, 63* *y)*
		Credibility/trustworthiness of source	14 (13.5)	*“It would depend whether it was a health professional I had had previous contact with … I would feel more comfortable receiving information from my own GP” (ID 282, F, 29* *y)*
		Pre-emptive family contact preferred	7 (8.0)	*“I would prefer to be told by my relative to expect the letter” (ID 563, F, 47* *y)*
		More information wanted	7 (8.0)	*“Who the relative was and how ill they were ie how they found out about the condition” (ID 563, M, 77* *y)*
		Emotional impact	3 (3.4)	*“I would worry immediately so having someone it discuss further is good” (ID 1005, F, 49* *y)*
		Benefits outweigh risks	1 (1.1)	*“Benefit/risk outweighs the concern” (ID 251, F, 29* *y)*
	Significant concerns (*n* = 40)	Use/provision of contact details without permission	13 (32.5)	*“This is an invasion of privacy. My relative has no right to give out my details to anyone” (ID 179, F, 32* *y)*
				*“My health details are of a very private matter to me. I would be furious if anyone did not respect that and sent me unsolicited mail about my health” (ID 801, M, 71* *y)*
		Use of personal information	10 (25.0)	*“I would be concerned this information could fall into a health or life insurers hands which could be detrimental financially” (ID 876, M, 62* *y)*
				*“I have little to no trust in those holding my information, even with all of the privacy legislation in place” (ID 597, M, 51* *y)*
		Privacy/confidentiality concern	7 (17.5)	*“Invasion of privacy” (ID 179, F, 32* *y)*
		Authenticity/possibility of scam	4 (10.0)	*“There is no naming of the relative to be able to confirm if this is legitimate or not” (ID 49, F, 63* *y)*
		Reliability of delivery method	1 (2.1)	*“If the letter was delivered to the wrong address or simply lost or stolen” (ID 183, F, 31* *y)*
		Pre-emptive family contact preferred	2 (5.0)	*“This information should come from the family member first” (ID 698, M, 70* *y)*
		Emotional impact	1 (2.5)	*“I would find it strange and alarming” (ID 1103, M, 47* *y)*
NON-PRIVACY (OTHER) CONCERNS (*n* = 60)	A little concern (*n* = 39)	Main categories	*n* (%)	Example quote/s
		Emotional impact/unwanted information	10 (25.6)	*“It would raise concerns; you can’t ‘unknow’ what you have just been told” (ID 96, F, 69* *y)*
				*“I would begin to worry and become anxious, no matter the outcome” (ID 926, F, 61* *y)*
		Use of personal information	6 (15.4)	*“What else happens with my personal information that’s been provided” (ID 1, F, 36* *y)*
		Possibility of letter being a scam	6 (15.4)	*“I would think that this coming out of the blue was probably a scam” (ID 38, M, 68* *y)*
		Reliability of delivery method	5 (12.8)	*“If the letter ended up in someone else’s mailbox (ID 1068, F, 37* *y”*
		Credibility/trustworthiness of source	5 (12.8)	*“Make sure it is legitimate and a contact number to verify the Doctor’s / Health Professional’s identity” (ID 843, M, 65* *y)*
		Use/provision of contact details without permission	4 (10.3)	*“My contact details should not be provided without my permission” (ID 1009, F, 49* *y)*
		Unreliability of family	1 (2.6)	*“Only that it relies on family member contact as well, and many family members can be less than reliable.” (ID 24, M, 31* *y)*
	Significant concerns (*n* = 21)	Invasion of privacy	8 (38.1)	*“Other family member/s will know about the letter and I would find that information too far outside my idea of health privacy. In other words, let me deal with my health on my own terms.” (ID 801, M, 71* *y)*
		Emotional impact/unwanted information	5 (23.8)	*“Confronting with information I may not want to know” (ID 156, F, 32* *y)*
		Possibility of letter being a scam/ marketing by commercial company	3 (14.3)	*“Just seems like the health professional is trying to get more business” (ID 36, M, 37* *y)*
		Use of personal information	2 (9.5)	*“Data security must [sic] needed” (ID 583, M, 42* *y)*
		Reliability of delivery method	1 (4.8)	*“Another member of my household may receive the letter and read him/herself” (ID 994, F, 37* *y)*

## Discussion

This study provides a snapshot of the Australian public’s views and preferences regarding direct contact by HPs about medically actionable genetic risk. Among individuals recruited outside the clinical genetics context, there was a high level of interest in genetic risk information and the opportunity to learn more about this area. Respondents expressed a preference for being told about their possible genetic risk, being contacted directly by a HP and being provided with specific information about the genetic condition up front. Few respondents had significant privacy concerns about this practice.

### Preferences about contact

A majority of respondents preferred to be contacted directly by a HP with genetic risk information, with only a sub-set wanting advance notification by a relative. A very small minority of respondents preferred to receive the letter from a relative, which is a significant finding given providing a “family letter” is the current practice in Australia. Further, some families with genetic variants have communication difficulties due to fragile family relations [32], members who are reluctant to disseminate risk information [4, 33] or who share it inappropriately [19]. In Australia, despite HPs routinely advising about the importance of informing family members, an estimated 20–40% of relatives don’t receive information about their potential genetic risk [9].

While some respondents preferred advance notice from relatives before direct contact by a HP, equally as many would rather not hear from family members, highlighting the varied and subjectively different experiences of families. This accords with research showing some individuals have poor relationships with relatives, and that reaching out to pass a letter to a relative is often complex and onerous [19] which can be a barrier to effective family communication [34, 35]. Despite some HPs expressing reservations about their ability to contact patients directly [36], or views that patients are better placed to communicate with relatives [19], this is often not true [4, 32]. Further, HPs often do not know whether relatives ultimately receive the information [37]. These findings underscore the importance of providing each patient with the best tools for disseminating risk information within their family, including offering direct notification by a HP where appropriate.

Respondents preferred to receive more specific information about the genetic variant in the family rather than less. Some respondents who initially preferred less information changed their mind after reviewing the draft letters, and some indicated they would like even more detailed information at first. This echoes other findings, which note the need to balance strong wording that could cause anxiety, and vague wording that doesn’t convey the seriousness of risk [19]. Further, vague wording could lead to misunderstanding and may also create anxiety. Our findings provide some basis for understanding the preferences of the general public (90% of respondents rated both letters easy to understand); however, the final content of such letters should undergo further testing with a range of consumers as well as clinicians.

Our findings accord with a recent survey of the Swedish general public (*n* = 977), of whom ~90% would want to know about potential genetic risk of colorectal cancer [38]. The majority (> 75%) preferred notification by a HP, with a minority preferring notification by a family member. A letter (closely followed by a phone call) was the preferred method of contact (38%). No specific questions about privacy (or non-privacy) concerns were included in the Swedish study, although participants could enter free text. The authors did not mention any particular privacy concerns arising. They did note Sweden’s specific socio-cultural context, which includes access to public healthcare and high trust in the healthcare system. Australia similarly has a publicly-funded healthcare system, meaning that access to testing for at-risk relatives should not incur out-of-pocket costs. Other studies in the US [18], UK [39], the Netherlands [11], Finland [13] and Australia [26, 27] have similarly found the majority of at-risk relatives support direct contact by health professionals.

### Privacy and non-privacy concerns

While most respondents in our study reported no privacy concerns about direct notification by HPs, a small minority had significant privacy concerns. Most of those also expressed strong non-privacy concerns, indicating a vocal minority with broad objections to the notion of information being shared with a HP to facilitate risk notification. General concerns about privacy can be barriers to communication about genetic risk within families in a minority of respondents, even where HPs are not directly involved [21, 35, 40]. Questions about whether local privacy regulations prohibit this practice have been raised and resolved internationally (for example in the Netherlands [41] and the US [42]). An analysis of Australian (federal and state/territory) privacy laws has recently been conducted by authors of this manuscript, and concludes that direct notification of relatives, with patient consent, can be undertaken in accordance with Australian privacy laws [43]. However, there is clearly a distinction between whether local privacy regulations permit a practice, and the public’s support of that practice. In our study, while a vocal minority of respondents had strong concerns about the use of personal contact details to inform relatives of genetic risk, the vast majority reported no privacy concerns with this practice.

It is important to note that the survey did not define “privacy” for the purposes of asking about privacy/non-privacy concerns, meaning respondents answered based on their understanding of the word’s meaning. Consequently, there was some overlap between the concepts expressed as privacy concerns and as non-privacy concerns. Beyond the vocal minority with significant concerns, a small number of respondents also expressed “a little concern” when asked about privacy/non-privacy concerns. Their comments are instructive, in considering how a direct approach to individuals would be best designed. Many of these concerns can be managed through adherence to privacy regulations and clear explanations in the initial contact letter. These include concerns about how genetic information is stored, shared and managed, concerns about whether the initial letter is a “scam” or marketing ploy, the need for reassurance of confidentiality, and access to follow-up support and resources. Concerns about the authenticity of electronic and hard copy communications are not surprising, given the current prevalence of online and other scams. Innovative methods for facilitating direct communication to at-risk relatives (such as those used for Covid-19 contact tracing [44]) in a trusted environment should be considered by policy-makers in future.

Other concerns, which are valid and are more difficult to address, include the potential use of genetic information by insurance companies. Genetic discrimination in life insurance is legal in Australia [45] and discrimination concerns are frequently reported by health professionals and consumers and are a known deterrent to genetic testing in Australia [46–49]. This issue requires urgent policy attention by the Australian government for consumer protection, but it does not apply differently to notification by relatives or directly by HPs. The main consideration for HPs in this regard is to advise relatives of potential insurance implications before genetic testing. Although this is already included in genetic counselling guidelines in Australia [2], family letters do not always contain information about insurance implications, which must be discussed, if relevant, before consent for testing is given.

The public health benefit of disseminating medically actionable genetic risk information to relatives is generally considered to outweigh minority concerns about the “right not to know” or the use of personal information for this purpose [41, 50]. Some commentators argue persuasively that HPs have a moral duty to make actionable genetic information available to at-risk relatives of patients, which cannot be obviated through merely encouraging moral information sharing by their patients [51]. One respondent in free-text comments with “a little concern” even recognised the benefits of knowing this risk information, noting that “benefit/risk outweighs the concern”. Similar findings emerged from a Canadian study [37], where a participant who had experienced sudden cardiac arrest before genetic testing stated, “you’d have to be an idiot not to be thankful for getting the information…[my family members] wouldn’t be worried about privacy if their health is a concern” (at 823). A participant in the same study compared direct contact in clinical genetics to common COVID-19 notification measures introduced during the pandemic for public health protection, stating, “especially now with this whole COVID thing, I wouldn’t mind getting notified by healthcare. Like, ‘hey, you might have been exposed to COVID.’ I see it in that way” (at 823). Considering methods of genetic notification in light of technology developed to assist with COVID-19 close contact notification in Australia would provide substantial practical insight.

Strengths of this study include its method of data collection. Research in areas such as genetics can be subject to responder bias, where individuals with prior interest in the topic choose to participate in research. Although this is still a potential limitation of all voluntary research, using an internationally recognised market research company likely enabled us to reach a more varied and representative sample of Australians than those who would ordinarily engage with genetics research. Limitations of the study included the survey length (6–10 mins maximum completion time), which constrained the level of detailed explanation that could be given. Further, it may be difficult to generalise our findings to members of the public in other countries with differing legal regulations and funding structures for healthcare and cascade genetic testing.

This study has provided considerable insight into the preferences and views of the Australian public regarding direct notification of at-risk relatives by HPs with patient consent. The majority of respondents expressed strong interest in being informed about medically actionable genetic risk, and did not have any privacy concerns about direct notification by a HP. The concerns raised by some respondents provide guidance regarding matters to be considered in the design of direct notification methodology. While the majority of respondents preferred a HP contact them rather than a relative, preferences varied regarding relatives’ involvement in the notification process, highlighting the need for flexible systems predicated on family circumstances. Additional research with Australian clinical and laboratory services is currently underway to understand their current practices and views regarding this issue, and the necessary considerations for guidelines in this area. Future research should focus on the development of guidelines and refining the methodology for direct contact of at-risk relatives, including the content and wording of proposed letters.

## Supplementary information

Supplementary file S1

Supplementary file S2

Supplementary file S3

Supplementary file S4

## Data Availability

Summary data are included within the manuscript and additional data are provided in supplementary files. Further additional data are available on reasonable request from the corresponding author.
