# Supplementary material for: Direct notification by health professionals of relatives at-risk of genetic conditions (with patient consent): views of the Australian public
Source: Eur J Hum Genet. 2023 Jun 6;32(1):98–108. doi: 10.1038/s41431-023-01395-9 (PMC10242214; doi:10.1038/s41431-023-01395-9)
Supplement: Supplementary file 3 — Supplementary file S3 [file 41431_2023_1395_MOESM3_ESM.pdf]

Notification of medically relevant genetic information

Genetic testing for certain DNA variants can tell us about high risks of developing health conditions in the future, like some cancers or cardiac conditions. Often these are conditions that can be prevented, or detected and treated early.

Because DNA is inherited (passed from parents to children), this risk can run in families. When one family member finds out about this type of risk, their managing health professional will usually advise them to tell their blood relatives about the possibility that they might also have the same DNA variants and the same health risks.

In this survey, we will ask for your thoughts about receiving this type of genetic information from a health professional. Don’t worry if you don’t know too much about this topic – we are interested in your views, no matter your level of knowledge.

Year of birth ______________________________

Sex
- ◯ Male
- ◯ Female
- ◯ Other

State of residence
- ◯ Australian Capital Territory
- ◯ New South Wales
- ◯ Northern Territory
- ◯ Queensland
- ◯ South Australia
- ◯ Tasmania
- ◯ Victoria
- ◯ Western Australia

I live in an area that is
- ◯ Rural
- ◯ Urban

Highest level of education attained
- ◯ Before year 12
- ◯ Year 12
- ◯ TAFE/diploma
- ◯ Undergraduate degree
- ◯ Post-graduate degree

What are your thoughts about being told (by a health professional) about genetic information that might show that you are at risk for future health problems that can be prevented (or detected and treated early)?

This is a hypothetical question – we will not contact you to offer you genetic information.

- ◯ I would want to be contacted and told that this information exists, and be given the option to find out more or not
- ◯ I would want to be contacted and told up-front what this information is and what the health risks to me are
- ◯ I would not want to be contacted to be offered this type of genetic information

Please read the letter below (Letter 1). Imagine you personally received this letter out of the blue – you had no prior warning about it or its contents.

Australian Clinical Genetics Service

Dear [name]

I am a medical specialist with the Australian clinical genetics service. I am writing to tell you about important health information that is significant for you and your family members.

A biological relative of yours has been tested by our service and found to have a DNA change that increases the risk of developing an inherited medical condition. Your relative has asked that we share this information with you.

You may have inherited the DNA change found in your relative. If so, you will be able to access preventative options to avoid developing the condition, or to detect and treat it early. We have not included the name of the condition because you have not yet asked for this information for yourself.

If you have inherited this DNA change, you may also pass it on to any biological children you have/may have in the future.

You can have a genetic test (at no cost to you) to find out whether:

1) you have inherited the DNA change and need extra health care, or
2) you have not inherited the DNA change and do not need extra health care.

We urge you to take this matter seriously. This information could be very important for the health of you and your close relatives. We recommend that you contact us, or discuss this matter with your doctor within the next few months.

What can you do if you would like a genetic test or have any questions?

You can contact us by phone or email on (03) 99999999 or info@geneticsinfo.com with any questions or to discuss the next steps. Please quote the reference number at the top of this letter.

If you do not want further information or an appointment at this stage, you are free to change your mind in the future. Please feel free to contact us at the above contact details at any time.

Yours sincerely

Dr G Netix
Clinical Geneticist
Australian Clinical Genetics Service

A note about privacy
Your relative provided us with your contact details so that we could give you this important information. We will only use your personal details for that purpose, and will not use them to contact you for other purposes. We will not provide your contact details to anyone else without your consent (unless required by law). We will delete or correct these details at your request, and you can contact us to request a copy of our privacy policy or to make a complaint about use of your information. Any information you provide to us will be treated confidentially. We will not tell anyone else, including your relatives, about any contact we may have with you without your permission. For the same reason, we cannot give you any information about other family members, including the relative who has provided us with your contact details.

In the hypothetical scenarios described in this survey, your family member would have provided your contact details to the health professional and asked them to send the letter directly to you. The health service would comply with privacy regulations and would not use your details for any other purpose.

You may have noticed that the letter (Letter 1) did not say which genetic condition your hypothetical family member's test relates to. Please select the option that best reflects your thoughts about this:

◯ I would have felt better informed if the genetic condition had been included in the letter
◯ Including the genetic condition in the letter would have been overwhelming for me at this early stage
◯ Other (please elaborate) ______

How easy was Letter 1 to understand?

◯ Very easy to understand
◯ Easy enough to understand
◯ A bit difficult to understand
◯ Very difficult to understand

Now read the letter below (Letter 2).

Australian Clinical Genetics Service

Dear [name]

I am a medical specialist with the Australian clinical genetics service. I am writing to tell you about important health information that is significant for you and your family members.

A biological relative of yours has been tested by our service and found to have a DNA change in the *BRCA1* gene. This gene change causes a considerably increased risk of developing certain cancers, including breast and ovarian cancer in women and prostate cancer in men. Your relative has asked that we share this information with you.

You may have inherited the DNA change found in your relative. If so, you will be able to access high-risk screening and other preventative options to avoid developing cancer, or to detect and treat it early.

If you have inherited this DNA change, you may also pass it on to any biological children you have/may have in the future.

You can have a genetic test (at no cost to you) to find out whether:

1) you have inherited the DNA change and need extra health care, or
2) you have not inherited the DNA change and do not need extra health care.

What can you do if you want a genetic test or have any questions?

You can contact us by phone or email on (03) 99999999 or info@geneticsinfo.com with any questions or to discuss the next steps. Please quote the reference number at the top of this letter.

If you do not want further information or an appointment at this stage, you are free to change your mind in the future. Please feel free to contact us at the above contact details at any time.

Yours sincerely

Dr G Netix
Clinical Geneticist
Australian Clinical Genetics Service

A note about privacy

Your relative provided us with your contact details so that we could give you this important information. We will only use your personal details for that purpose, and will not use them to contact you for other purposes. We will not provide your contact details to anyone else without your consent (unless required by law). We will delete or correct these details at your request, and you can contact us to request a copy of our privacy policy or to make a complaint about use of your information. Any information you provide to us will be treated confidentially. We will not tell anyone else, including your relatives, about any contact we may have with you without your permission. For the same reason, we cannot give you any information about other family members, including the relative who has provided us with your contact details.

This letter (Letter 2) is similar to Letter 1 but contains more information about the genetic condition. Please select the option that best reflects your thoughts about this:

◯ I felt better informed from this letter than from the previous letter
◯ It felt overwhelming to have that level of information at this early stage
◯ Other (please elaborate) ______

How easy was Letter 2 to understand?

◯ Very easy to understand
◯ Easy enough to understand
◯ A bit difficult to understand
◯ Very difficult to understand

Having read both letters, which letter do you prefer?

◯ Letter 1 (no specific information about the genetic condition)
◯ Letter 2 (specific information about the genetic condition)
◯ No preference

Do you have any other comments about the two letters - for example, were there any aspects of either letter that you weren't comfortable with, or that you particularly liked?

If you had the option to choose how you received this letter, which would you prefer?

◯ A health professional sent me the letter directly
◯ My family member provided me with the letter
◯ A health professional sent me the letter directly, but my family member also contacted me to explain or prepare me
◯ It would depend on who the family member was as to whether I would prefer to be contacted by them or a health professional
◯ I wouldn't mind whether a family member or a health professional provided me with the letter
◯ I would prefer not to be told this information at all

Do you have any comments (optional)?

Would you have any privacy concerns about being sent a letter directly by a health professional, using details provided to them by your relative?

◯ No concerns
◯ A little concern (please elaborate) ______
◯ Significant concerns (please elaborate) ______

Would you have any other (non-privacy related) concerns about being sent a letter directly in this way?

◯ No concerns
◯ A little concern (please elaborate) ______
◯ Significant concerns (please elaborate) ______

There are several ways of contacting family members who are unaware of their risk with this type of genetic information. We have shown you a letter, which is one way. Which of these ways of contacting family members do you think would be ok for a health professional to use?

Select all that apply

☐ A phone call
☐ A text message
☐ A letter (as shown above) by post
☐ A letter by email
☐ None

Which of these ways of being contacted by a health professional would you prefer?

◯ A phone call
◯ A text message
◯ A letter by post
◯ A letter by email
◯ None
◯ No preference

If a health professional contacted you in one of these ways, would you prefer that your family member contacted you first, to let you know that it was coming?

◯ Yes
◯ No
◯ Unsure

If you answered [cs_fam_contact_preference], please provide an optional comment if you'd like.

Do you have any final comments about any of the matters raised in this questionnaire?
