# Supplementary material for: Direct notification by health professionals of relatives at-risk of genetic conditions (with patient consent): views of the Australian public
Source: Eur J Hum Genet. 2023 Jun 6;32(1):98–108. doi: 10.1038/s41431-023-01395-9 (PMC10242214; doi:10.1038/s41431-023-01395-9)
Supplement: Supplementary file 4 — Supplementary file S4 [file 41431_2023_1395_MOESM4_ESM.pdf]

```
  ___  ____  ____  ____  ____(R)
 /__    /   ____/   /   ____/
___/   /   /___/   /   /___/
  Statistics/Data analysis

    User: Jane Tiller
 Project: at-risk notification
```

```
      name:  <unnamed>
       log:  /Users/keri.finlay/Documents/Stata/logfile_survey.smcl
  log type:  smcl
 opened on:  15 Sep 2022, 13:46:19

 1 . do "/Users/keri.finlay/Documents/Stata/ConsumerSurveyDynata-ForAnalysis_STATA_2022-08-24_1003 (1).do"

 2 . * Name: Keri FInlay
 3 . * Date: Aug 2022
 4 . * Purpose: Data Analysis of consumer survey
 5 .
 6 .
 7 . *change directory
 8 . cd "/Users/keri.finlay/Documents/Stata/"
   /Users/keri.finlay/Documents/Stata

 9 .
10 . version 13

11 . clear

12 .
13 . import delimited record_id redcap_survey_identifier notification_of_medi_v_0 cs_birth_year cs_sex cs_state cs_area_t
   > s_edu cs_thoughts cs_letter1_thoughts cs_letter1_thoughts_other cs_letter1_difficulty cs_letter2_thoughts cs_letter2
   > ghts_other cs_letter2_difficulty cs_letter_preference cs_letters_comments cs_contact_preference cs_contact_pref_comm
   > s_privacy_concerns cs_privacy_concerns_little cs_privacy_concerns_signif cs_non_priv_concerns cs_non_priv_concerns_s
   >  cs_non_priv_concerns_little cs_contact_ways cs_contact_ways_preference cs_fam_contact_preference cs_fam_contact_pre
   > es cs_general_comments notification_of_medi_v_1 using "/Users/keri.finlay/Documents/Stata/ConsumerSurveyDynata-ForAn
   > s_DATA_NOHDRS_2022-08-24_1003.csv", varnames(nonames)
   (31 vars, 1,030 obs)

14 .
15 . label data "ConsumerSurveyDynata-ForAnalysis_DATA_NOHDRS_2022-08-24_1003.csv"

16 .
17 . label define cs_sex_ 1 "Male" 2 "Female" 999 "Other"

18 . label define cs_state_ 1 "Australian Capital Territory" 2 "New South Wales" 3 "Northern Territory" 4 "Queensland" 5
   > h Australia" 6 "Tasmania" 7 "Victoria" 8 "Western Australia"

19 . label define cs_area_type_ 1 "Rural" 2 "Urban"

20 . label define cs_edu_ 1 "Before year 12" 2 "Year 12" 3 "TAFE/diploma" 4 "Undergraduate degree" 5 "Post-graduate degre

21 . label define cs_thoughts_ 1 "I would want to be contacted and told that this information exists, and be given the op
   > to find out more or not" 2 "I would want to be contacted and told up-front what this information is and what the hea
   > isks to me are" 3 "I would not want to be contacted to be offered this type of genetic information"

22 . label define cs_letter1_thoughts_ 1 "I would have felt better informed if the genetic condition had been included in
   > letter" 2 "Including the genetic condition in the letter would have been overwhelming for me at this early stage" 3
   > r (please elaborate) {cs_letter1_thoughts_other}"

23 . label define cs_letter1_difficulty_ 1 "Very easy to understand" 2 "Easy enough to understand" 3 "A bit difficult to
   > stand" 4 "Very difficult to understand"

24 . label define cs_letter2_thoughts_ 1 "I felt better informed from this letter than from the previous letter" 2 "It fe
   > erwhelming to have that level of information at this early stage" 3 "Other (please elaborate) {cs_letter2_thoughts_o
   > "

25 . label define cs_letter2_difficulty_ 1 "Very easy to understand" 2 "Easy enough to understand" 3 "A bit difficult to
   > stand" 4 "Very difficult to understand"
```

```
 26 . label define cs_letter_preference_ 1 "Letter 1 (no specific information about the genetic condition)" 2 "Letter 2 (s
    > ic information about the genetic condition)" 3 "No preference"

 27 . label define cs_contact_preference_ 1 "A health professional sent me the letter directly" 2 "My family member provid
    >  with the letter" 3 "A health professional sent me the letter directly, but my family member also contacted me to ex
    >  or prepare me" 4 "It would depend on who the family member was as to whether I would prefer to be contacted by them
    >  health professional" 5 "I wouldnt mind whether a family member or a health professional provided me with the letter
    > I would prefer not to be told this information at all"

 28 . label define cs_privacy_concerns_ 1 "No concerns" 2 "A little concern (please elaborate) {cs_privacy_concerns_little
    > "Significant concerns (please elaborate) {cs_privacy_concerns_signif}"

 29 . label define cs_non_priv_concerns_ 1 "No concerns" 2 "A little concern (please elaborate) {cs_non_priv_concerns_litt
    > 3 "Significant concerns (please elaborate) {cs_non_priv_concerns_signif}"

 30 . label define cs_contact_ways_preference_ 1 "A phone call" 2 "A text message" 3 "A letter by post" 4 "A letter by ema
    >  "None" 100 "No preference"

 31 . label define cs_fam_contact_preference_ 1 "Yes" 0 "No" 2 "Unsure"

 32 . label define notification_of_medi_v_1_ 0 "Incomplete" 1 "Unverified" 2 "Complete"

 33 .
 34 . label values cs_sex cs_sex_

 35 . label values cs_state cs_state_

 36 . label values cs_area_type cs_area_type_

 37 . label values cs_edu cs_edu_

 38 . label values cs_thoughts cs_thoughts_

 39 . label values cs_letter1_thoughts cs_letter1_thoughts_

 40 . label values cs_letter1_difficulty cs_letter1_difficulty_

 41 . label values cs_letter2_thoughts cs_letter2_thoughts_

 42 . label values cs_letter2_difficulty cs_letter2_difficulty_

 43 . label values cs_letter_preference cs_letter_preference_

 44 . label values cs_contact_preference cs_contact_preference_

 45 . label values cs_privacy_concerns cs_privacy_concerns_

 46 . label values cs_non_priv_concerns cs_non_priv_concerns_

 47 . label values cs_contact_ways_preference cs_contact_ways_preference_

 48 . label values cs_fam_contact_preference cs_fam_contact_preference_

 49 . label values notification_of_medi_v_1 notification_of_medi_v_1_

 50 .
 51 .
 52 .
 53 . label variable record_id "Record ID"

 54 . label variable redcap_survey_identifier "Survey Identifier"
```

```
55 . label variable notification_of_medi_v_0 "Survey Timestamp"

56 . label variable cs_birth_year "Year of birth"

57 . label variable cs_sex "Sex"

58 . label variable cs_state "State of residence"

59 . label variable cs_area_type "I live in an area that is"

60 . label variable cs_edu "Highest level of education attained "

61 . label variable cs_thoughts "What are your thoughts about being told (by a health professional) about genetic informa
   > that might show that you are at risk for future health problems that can be prevented (or detected and treated early
   > is is a hypothetical question – we will not contact you to offer you genetic information."
   note: label truncated to 80 characters

62 . label variable cs_letter1_thoughts "You may have noticed that the letter (Letter 1) did not say which genetic condit
   > our hypothetical family member’s test relates to. Please select the option that best reflects your thoughts about th
   note: label truncated to 80 characters

63 . label variable cs_letter1_thoughts_other "Please elaborate"

64 . label variable cs_letter1_difficulty "How easy was Letter 1 to understand?"

65 . label variable cs_letter2_thoughts "This letter (Letter 2) is similar to Letter 1 but contains more information abou
   >  genetic condition. Please select the option that best reflects your thoughts about this:"
   note: label truncated to 80 characters

66 . label variable cs_letter2_thoughts_other "Please elaborate"

67 . label variable cs_letter2_difficulty "How easy was Letter 2 to understand?"

68 . label variable cs_letter_preference "Having read both letters, which letter do you prefer? "

69 . label variable cs_letters_comments "Do you have any other comments about the two letters - for example, were there a
   > pects of either letter that you werent comfortable with, or that you particularly liked? "
   note: label truncated to 80 characters

70 . label variable cs_contact_preference "If you had the option to choose how you received this letter, which would you 
   > r?"
   note: label truncated to 80 characters

71 . label variable cs_contact_pref_comment "Do you have any comments (optional)?"

72 . label variable cs_privacy_concerns "Would you have any privacy concerns about being sent a letter directly by a heal
   > ofessional, using details provided to them by your relative? "
   note: label truncated to 80 characters

73 . label variable cs_privacy_concerns_little "Please elaborate"

74 . label variable cs_privacy_concerns_signif "Please elaborate"

75 . label variable cs_non_priv_concerns "Would you have any other (non-privacy related) concerns about being sent a lett
   > rectly in this way?"
   note: label truncated to 80 characters

76 . label variable cs_non_priv_concerns_signif "Please elaborate"

77 . label variable cs_non_priv_concerns_little "Please elaborate"

78 . label variable cs_contact_ways "There are several ways of contacting family members who are unaware of their risk wi
   > is type of genetic information. We have shown you a letter, which is one way. Which of these ways of contacting fami
   > mbers do you think would be ok for a health professional to use?Select all that apply"
   note: label truncated to 80 characters
```

```
79 . label variable cs_contact_ways_preference "Which of these ways of being contacted by a health professional would you
   > er?"
   note: label truncated to 80 characters

80 . label variable cs_fam_contact_preference "If a health professional contacted you in one of these ways, would you pre
   > hat your family member contacted you first, to let you know that it was coming?"
   note: label truncated to 80 characters

81 . label variable cs_fam_contact_pref_notes "If you answered [cs_fam_contact_preference], please provide an optional co
   >  if youd like."
   note: label truncated to 80 characters

82 . label variable cs_general_comments "Do you have any final comments about any of the matters raised in this questionn
   >  "
   note: label truncated to 80 characters

83 . label variable notification_of_medi_v_1 "Complete?"

84 .
85 . order record_id redcap_survey_identifier notification_of_medi_v_0 cs_birth_year cs_sex cs_state cs_area_type cs_edu
   > oughts cs_letter1_thoughts cs_letter1_thoughts_other cs_letter1_difficulty cs_letter2_thoughts cs_letter2_thoughts_o
   > cs_letter2_difficulty cs_letter_preference cs_letters_comments cs_contact_preference cs_contact_pref_comment cs_priv
   > oncerns cs_privacy_concerns_little cs_privacy_concerns_signif cs_non_priv_concerns cs_non_priv_concerns_signif cs_no
   > v_concerns_little cs_contact_ways cs_contact_ways_preference cs_fam_contact_preference cs_fam_contact_pref_notes cs_
   > al_comments notification_of_medi_v_1

86 . set more off

87 . describe

   Contains data
    Observations:         1,030                  ConsumerSurveyDynata-ForAnalysis_DATA_NOHDRS_2022-08-24_1003.csv
       Variables:            31
   ------------------------------------------------------------------------------------------------------------------
   Variable      Storage   Display    Value
       name         type    format    label      Variable label
   ------------------------------------------------------------------------------------------------------------------
   record_id       int     %8.0g                 Record ID
   redcap_survey~r byte    %8.0g                 Survey Identifier
   notification_~0 str19   %19s                  Survey Timestamp
   cs_birth_year   int     %8.0g                 Year of birth
   cs_sex          int     %8.0g      cs_sex_    Sex
   cs_state        byte    %28.0g     cs_state_
                                                 State of residence
   cs_area_type    byte    %8.0g      cs_area_type_
                                                 I live in an area that is
   cs_edu          byte    %20.0g     cs_edu_    Highest level of education attained
   cs_thoughts     byte    %115.0g    cs_thoughts_
                                                 What are your thoughts about being told (by a health professional) about
                                                   genetic
   cs_letter1_th~s byte    %101.0g    cs_letter1_thoughts_
                                                 You may have noticed that the letter (Letter 1) did not say which geneti
                                                   condit
   cs_letter1_th~r str636  %636s                 Please elaborate
   cs_letter1_di~y byte    %29.0g     cs_letter1_difficulty_
                                                 How easy was Letter 1 to understand?
   cs_letter2_th~s byte    %74.0g     cs_letter2_thoughts_
                                                 This letter (Letter 2) is similar to Letter 1 but contains more informat
                                                   abou
   cs_letter2_th~r str636  %636s                 Please elaborate
   cs_letter2_di~y byte    %29.0g     cs_letter2_difficulty_
                                                 How easy was Letter 2 to understand?
   cs_letter_pre~e byte    %62.0g     cs_letter_preference_
                                                 Having read both letters, which letter do you prefer?
   cs_letters_co~s str754  %754s                 Do you have any other comments about the two letters - for example, were
                                                   there a
   cs_contact_pr~e byte    %122.0g    cs_contact_preference_
                                                 If you had the option to choose how you received this letter, which woul
                                                   pr
   cs_contact_pr~t str753  %753s                 Do you have any comments (optional)?
   cs_privacy_co~s byte    %68.0g     cs_privacy_concerns_
                                                 Would you have any privacy concerns about being sent a letter directly b
                                                   heal
   cs_privacy_co~e str218  %218s                 Please elaborate
```

```
cs_privacy_co~f str307  %307s                  Please elaborate
cs_non_priv_c~s byte    %69.0g      cs_non_priv_concerns_
                                               Would you have any other (non-privacy related) concerns about being sent
                                                 lette
cs_non_priv_c~f str190  %190s                  Please elaborate
cs_non_priv_c~e str193  %193s                  Please elaborate
cs_contact_ways str7    %9s                    There are several ways of contacting family members who are unaware of t
                                                 ris
cs_contact_wa~e byte    %17.0g      cs_contact_ways_preference_
                                               Which of these ways of being contacted by a health professional would yo
                                                 prefer
cs_fam_contac~e byte    %8.0g       cs_fam_contact_preference_
                                               If a health professional contacted you in one of these ways, would you p
                                                 th
cs_fam_contac~s str387  %387s                  If you answered [cs_fam_contact_preference], please provide an optional
                                                 comment
cs_general_co~s str439  %439s                  Do you have any final comments about any of the matters raised in this
                                                 questionn
notification_~1 byte    %10.0g      notification_of_medi_v_1_
                                               Complete?
```

Sorted by:
     Note: Dataset has changed since last saved.

```
88 .
89 . ************************************
90 . *Data Analysis
91 .
92 . **Descriptive stats***
93 .
94 . replace cs_birth_year = . if record_id == 404
   (1 real change made, 1 to missing)

95 . generate currentyear = 2023

96 . generate  age = currentyear - cs_birth_year
   (23 missing values generated)

97 . tab age, missing
```

age	Freq.	Percent	Cum.
18	1	0.10	0.10
19	5	0.49	0.58
20	10	0.97	1.55
21	13	1.26	2.82
22	10	0.97	3.79
23	26	2.52	6.31
24	20	1.94	8.25
25	15	1.46	9.71
26	15	1.46	11.17
27	16	1.55	12.72
28	12	1.17	13.88
29	14	1.36	15.24
30	10	0.97	16.21
31	18	1.75	17.96
32	18	1.75	19.71
33	29	2.82	22.52
34	26	2.52	25.05
35	24	2.33	27.38
36	19	1.84	29.22
37	14	1.36	30.58
38	26	2.52	33.11
39	21	2.04	35.15
40	14	1.36	36.50
41	29	2.82	39.32
42	26	2.52	41.84
43	25	2.43	44.27
44	19	1.84	46.12
45	14	1.36	47.48
46	21	2.04	49.51
47	19	1.84	51.36
48	14	1.36	52.72
49	20	1.94	54.66

50	21	2.04	56.70
51	19	1.84	58.54
52	17	1.65	60.19
53	20	1.94	62.14
54	20	1.94	64.08
55	18	1.75	65.83
56	13	1.26	67.09
57	16	1.55	68.64
58	13	1.26	69.90
59	17	1.65	71.55
60	15	1.46	73.01
61	6	0.58	73.59
62	14	1.36	74.95
63	19	1.84	76.80
64	19	1.84	78.64
65	16	1.55	80.19
66	17	1.65	81.84
67	11	1.07	82.91
68	17	1.65	84.56
69	8	0.78	85.34
70	16	1.55	86.89
71	10	0.97	87.86
72	11	1.07	88.93
73	16	1.55	90.49
74	14	1.36	91.84
75	7	0.68	92.52
76	8	0.78	93.30
77	9	0.87	94.17
78	7	0.68	94.85
79	7	0.68	95.53
80	9	0.87	96.41
81	2	0.19	96.60
82	1	0.10	96.70
83	1	0.10	96.80
84	3	0.29	97.09
85	2	0.19	97.28
86	1	0.10	97.38
87	3	0.29	97.67
88	1	0.10	97.77
.	23	2.23	100.00
Total	1,030	100.00	

```
 98 .
 99 . generate cs_age_range = age
    (23 missing values generated)

100 . recode cs_age_range(18/25 = 1) (26/40 = 2) (41/60 = 3) (61/80 = 4) (81/max = 5)
    (1007 changes made to cs_age_range)

101 . label define age_lbl 1 "18-24" 2 "25-40" 3 "41-60" 4 "61-80" 5 ">81"

102 . label values cs_age_range age_lbl

103 . mean age
```

Mean estimation   Number of obs = **1,007**

	Mean	Std. err.	[95% conf.	interval]
age	47.74479	.5231878	46.71812	48.77145

```
104 .
105 . tab cs_age_range
```

cs_age_range	Freq.	Percent	Cum.
18-24	100	9.93	9.93
25-40	276	27.41	37.34
41-60	376	37.34	74.68
61-80	241	23.93	98.61
>81	14	1.39	100.00
Total	1,007	100.00	

```
106 .
107 . tab cs_sex
```

Sex	Freq.	Percent	Cum.
Male	502	49.22	49.22
Female	514	50.39	99.61
Other	4	0.39	100.00
Total	1,020	100.00	

```
108 . *graph pie, over(cs_sex)
109 . *graph export "graphs/pie_sex.png", as(png) name("Graph") replace
110 .
111 . tab cs_state
```

State of residence	Freq.	Percent	Cum.
Australian Capital Territory	22	2.20	2.20
New South Wales	325	32.50	34.70
Northern Territory	2	0.20	34.90
Queensland	201	20.10	55.00
South Australia	81	8.10	63.10
Tasmania	20	2.00	65.10
Victoria	254	25.40	90.50
Western Australia	95	9.50	100.00
Total	1,000	100.00	

```
112 . *graph pie, over(cs_state)
113 . *graph export "graphs/pie_state.png", as(png) name("Graph") replace
114 .
115 . tab cs_area_type
```

I live in an area that is	Freq.	Percent	Cum.
Rural	263	26.17	26.17
Urban	742	73.83	100.00
Total	1,005	100.00	

```
116 . *graph pie, over(cs_area_type)
117 . *graph export "graphs/bar_area.png", as(png) name("Graph") replace
118 .
119 . tab cs_edu
```

Highest level of education attained	Freq.	Percent	Cum.
Before year 12	135	13.18	13.18
Year 12	186	18.16	31.35
TAFE/diploma	327	31.93	63.28
Undergraduate degree	247	24.12	87.40
Post-graduate degree	129	12.60	100.00
Total	1,024	100.00	

```
120 . *graph bar (count), over(cs_edu)
121 . *graph export "graphs/bar_edu.png", as(png) name("Graph") replace
122 .
123 . tab cs_thoughts
```

What are your thoughts about being told (by a health professional) about genetic	Freq.	Percent	Cum.
I would want to be contacted and told t	496	48.16	48.16
I would want to be contacted and told u	376	36.50	84.66
I would not want to be contacted to be	158	15.34	100.00
Total	1,030	100.00	

```
124 .
125 . tab cs_letter1_thoughts
```

You may have noticed that the letter (Letter 1) did not say which genetic condit	Freq.	Percent	Cum.
I would have felt better informed if th	749	72.72	72.72
Including the genetic condition in the	250	24.27	96.99
Other (please elaborate) {cs_letter1_th	31	3.01	100.00
Total	1,030	100.00	

```
126 .
127 . tab cs_letter1_difficulty
```

How easy was Letter 1 to understand?	Freq.	Percent	Cum.
Very easy to understand	428	41.55	41.55
Easy enough to understand	497	48.25	89.81
A bit difficult to understand	89	8.64	98.45
Very difficult to understand	16	1.55	100.00
Total	1,030	100.00	

```
128 .
129 . tab cs_letter2_thoughts
```

This letter (Letter 2) is similar to Letter 1 but contains more information abou	Freq.	Percent	Cum.
I felt better informed from this letter	775	75.24	75.24
It felt overwhelming to have that level	231	22.43	97.67
Other (please elaborate) {cs_letter2_th	24	2.33	100.00
Total	1,030	100.00	

```
130 .
131 . tab cs_letter2_difficulty
```

How easy was Letter 2 to understand?	Freq.	Percent	Cum.
Very easy to understand	454	44.08	44.08
Easy enough to understand	473	45.92	90.00
A bit difficult to understand	91	8.83	98.83
Very difficult to understand	12	1.17	100.00
Total	1,030	100.00	

132 .
133 . tab cs_letter_preference

Having read both letters, which letter do you prefer?	Freq.	Percent	Cum.
Letter 1 (no specific information about	217	21.07	21.07
Letter 2 (specific information about th	691	67.09	88.16
No preference	122	11.84	100.00
Total	1,030	100.00	

134 .
135 . tab cs_contact_preference

If you had the option to choose how you received this letter, which would you pr	Freq.	Percent	Cum.
A health professional sent me the lette	467	45.34	45.34
My family member provided me with the l	87	8.45	53.79
A health professional sent me the lette	237	23.01	76.80
It would depend on who the family membe	106	10.29	87.09
I wouldnt mind whether a family member	91	8.83	95.92
I would prefer not to be told this info	42	4.08	100.00
Total	1,030	100.00	

136 .
137 . tab cs_privacy_concerns

Would you have any privacy concerns about being sent a letter directly by a heal	Freq.	Percent	Cum.
No concerns	878	85.24	85.24
A little concern (please elaborate) {cs	104	10.10	95.34
Significant concerns (please elaborate)	48	4.66	100.00
Total	1,030	100.00	

138 .
139 . tab cs_non_priv_concerns

Would you have any other (non-privacy related) concerns about being sent a lette	Freq.	Percent	Cum.
No concerns	946	91.84	91.84
A little concern (please elaborate) {cs	57	5.53	97.38
Significant concerns (please elaborate)	27	2.62	100.00
Total	1,030	100.00	

140 .
141 . tab cs_contact_ways

There are several ways of contacting family members who are unaware of their ris	Freq.	Percent	Cum.
0	54	5.24	5.24
1	121	11.75	16.99
1,2	23	2.23	19.22
1,2,3	10	0.97	20.19
1,2,3,4	39	3.79	23.98
1,2,4	3	0.29	24.27
1,3	81	7.86	32.14

1,3,4	**64**	**6.21**	**38.35**
1,4	**26**	**2.52**	**40.87**
2	**54**	**5.24**	**46.12**
2,3	**5**	**0.49**	**46.60**
2,3,4	**16**	**1.55**	**48.16**
2,4	**8**	**0.78**	**48.93**
3	**303**	**29.42**	**78.35**
3,4	**121**	**11.75**	**90.10**
4	**102**	**9.90**	**100.00**
Total	**1,030**	**100.00**	

142 .
143 . tab cs_contact_ways_preference

Which of these ways of being contacted by a health professional would you prefer	Freq.	Percent	Cum.
None	**42**	**4.08**	**4.08**
A phone call	**220**	**21.36**	**25.44**
A text message	**72**	**6.99**	**32.43**
A letter by post	**418**	**40.58**	**73.01**
A letter by email	**240**	**23.30**	**96.31**
No preference	**38**	**3.69**	**100.00**
Total	**1,030**	**100.00**	

144 .
145 .
146 .
147 . ***************************Cross tabs****************************
148 .
149 . tab cs_thoughts cs_state, col

Key
frequency<br>*column percentage*

What are your thoughts about being told (by a health professional) about genetic	State of residence<br>Australia	New South	Northern	Queenslan	South Aus	Tasmania	Victoria	Western A	T
I would want to be co	**10**<br>**45.45**	**149**<br>**45.85**	**1**<br>**50.00**	**90**<br>**44.78**	**43**<br>**53.09**	**12**<br>**60.00**	**129**<br>**50.79**	**46**<br>**48.42**	 **4**
I would want to be co	**9**<br>**40.91**	**113**<br>**34.77**	**1**<br>**50.00**	**82**<br>**40.80**	**31**<br>**38.27**	**7**<br>**35.00**	**92**<br>**36.22**	**32**<br>**33.68**	 **3**
I would not want to b	**3**<br>**13.64**	**63**<br>**19.38**	**0**<br>**0.00**	**29**<br>**14.43**	**7**<br>**8.64**	**1**<br>**5.00**	**33**<br>**12.99**	**17**<br>**17.89**	 **1**
Total	**22**<br>**100.00**	**325**<br>**100.00**	**2**<br>**100.00**	**201**<br>**100.00**	**81**<br>**100.00**	**20**<br>**100.00**	**254**<br>**100.00**	**95**<br>**100.00**	**1**<br>**10**

150 . tab cs_thoughts cs_area_type, col

Key
frequency<br>*column percentage*

What are your thoughts about being told (by a health professional) about genetic	I live in an area that is Rural	Urban	Total
I would want to be co	**117**<br>**44.49**	**365**<br>**49.19**	**482**<br>**47.96**
I would want to be co	**102**<br>**38.78**	**269**<br>**36.25**	**371**<br>**36.92**
I would not want to b	**44**<br>**16.73**	**108**<br>**14.56**	**152**<br>**15.12**
Total	**263**<br>**100.00**	**742**<br>**100.00**	**1,005**<br>**100.00**

151 .
152 .
153 . tab cs_letter1_thoughts cs_sex, col

Key
frequency<br>*column percentage*

You may have noticed that the letter (Letter 1) did not say which genetic condit	Male	Sex Female	Other	Total
I would have felt bet	**372**<br>**74.10**	**366**<br>**71.21**	**3**<br>**75.00**	**741**<br>**72.65**
Including the genetic	**109**<br>**21.71**	**138**<br>**26.85**	**1**<br>**25.00**	**248**<br>**24.31**
Other (please elabora	**21**<br>**4.18**	**10**<br>**1.95**	**0**<br>**0.00**	**31**<br>**3.04**
Total	**502**<br>**100.00**	**514**<br>**100.00**	**4**<br>**100.00**	**1,020**<br>**100.00**

154 .
155 .
156 . tab cs_letter1_difficulty cs_edu, col

Key
frequency<br>*column percentage*

How easy was Letter 1 to understand?	Highest level of education attained Before ye	Year 12	TAFE/dipl	Undergrad	Post-grad	Total
Very easy to understa	50<br>37.04	70<br>37.63	142<br>43.43	107<br>43.32	55<br>42.64	424<br>41.41
Easy enough to unders	68<br>50.37	97<br>52.15	158<br>48.32	118<br>47.77	55<br>42.64	496<br>48.44
A bit difficult to un	17<br>12.59	14<br>7.53	21<br>6.42	21<br>8.50	15<br>11.63	88<br>8.59
Very difficult to und	0<br>0.00	5<br>2.69	6<br>1.83	1<br>0.40	4<br>3.10	16<br>1.56
Total	135<br>100.00	186<br>100.00	327<br>100.00	247<br>100.00	129<br>100.00	1,024<br>100.00

157 . tab cs_letter2_difficulty cs_edu, col

Key
frequency<br>*column percentage*

How easy was Letter 2 to understand?	Highest level of education attained Before ye	Year 12	TAFE/dipl	Undergrad	Post-grad	Total
Very easy to understa	56<br>41.48	76<br>40.86	144<br>44.04	109<br>44.13	66<br>51.16	451<br>44.04
Easy enough to unders	68<br>50.37	86<br>46.24	153<br>46.79	115<br>46.56	49<br>37.98	471<br>46.00
A bit difficult to un	11<br>8.15	22<br>11.83	26<br>7.95	21<br>8.50	10<br>7.75	90<br>8.79
Very difficult to und	0<br>0.00	2<br>1.08	4<br>1.22	2<br>0.81	4<br>3.10	12<br>1.17
Total	135<br>100.00	186<br>100.00	327<br>100.00	247<br>100.00	129<br>100.00	1,024<br>100.00

158 . tab cs_letter1_difficulty cs_letter2_difficulty, col

Key
frequency<br>*column percentage*

How easy was Letter 1 to understand?	How easy was Letter 2 to understand? Very easy	Easy enou	A bit dif	Very diff	Total
Very easy to understa	365<br>80.40	58<br>12.26	5<br>5.49	0<br>0.00	428<br>41.55
Easy enough to unders	80<br>17.62	382<br>80.76	33<br>36.26	2<br>16.67	497<br>48.25
A bit difficult to un	8<br>1.76	31<br>6.55	46<br>50.55	4<br>33.33	89<br>8.64
Very difficult to und	1<br>0.22	2<br>0.42	7<br>7.69	6<br>50.00	16<br>1.55
Total	454<br>100.00	473<br>100.00	91<br>100.00	12<br>100.00	1,030<br>100.00

```
159 .
160 .
161 . tab  cs_letter_preference cs_edu,col
```

Key
frequency *column percentage*

Having read both letters, which letter do you prefer?	Highest level of education attained Before ye	Year 12	TAFE/dipl	Undergrad	Post-grad	Total
Letter 1 (no specific	**28** **20.74**	**36** **19.35**	**74** **22.63**	**52** **21.05**	**26** **20.16**	**216** **21.09**
Letter 2 (specific in	**85** **62.96**	**129** **69.35**	**214** **65.44**	**175** **70.85**	**85** **65.89**	**688** **67.19**
No preference	**22** **16.30**	**21** **11.29**	**39** **11.93**	**20** **8.10**	**18** **13.95**	**120** **11.72**
Total	**135** **100.00**	**186** **100.00**	**327** **100.00**	**247** **100.00**	**129** **100.00**	**1,024** **100.00**

```
162 . tab cs_letter_preference cs_sex, col
```

Key
frequency *column percentage*

Having read both letters, which letter do you prefer?	Sex Male	Female	Other	Total
Letter 1 (no specific	**102** **20.32**	**113** **21.98**	**1** **25.00**	**216** **21.18**
Letter 2 (specific in	**336** **66.93**	**346** **67.32**	**2** **50.00**	**684** **67.06**
No preference	**64** **12.75**	**55** **10.70**	**1** **25.00**	**120** **11.76**
Total	**502** **100.00**	**514** **100.00**	**4** **100.00**	**1,020** **100.00**

```
163 . tab cs_letter_preference cs_age_range, col
```

Key
frequency *column percentage*

Having read both letters, which letter do you prefer?	cs_age_range 18-24	25-40	41-60	61-80	>81	Total
Letter 1 (no specific	**26** **26.00**	**70** **25.36**	**80** **21.28**	**32** **13.28**	**1** **7.14**	**209** **20.75**
Letter 2 (specific in	**63** **63.00**	**181** **65.58**	**245** **65.16**	**178** **73.86**	**11** **78.57**	**678** **67.33**
No preference	**11** **11.00**	**25** **9.06**	**51** **13.56**	**31** **12.86**	**2** **14.29**	**120** **11.92**

Total	100<br>100.00	276<br>100.00	376<br>100.00	241<br>100.00	14<br>100.00	1,007<br>100.00

164 .
165 . tab cs_privacy_concerns cs_edu, col

Key
frequency<br>*column percentage*

Would you have any privacy concerns about being sent a letter directly by a heal	Highest level of education attained<br>Before ye	Year 12	TAFE/dipl	Undergrad	Post-grad	Total
No concerns	122<br>90.37	163<br>87.63	289<br>88.38	197<br>79.76	101<br>78.29	872<br>85.16
A little concern (ple	8<br>5.93	19<br>10.22	29<br>8.87	32<br>12.96	16<br>12.40	104<br>10.16
Significant concerns	5<br>3.70	4<br>2.15	9<br>2.75	18<br>7.29	12<br>9.30	48<br>4.69
Total	135<br>100.00	186<br>100.00	327<br>100.00	247<br>100.00	129<br>100.00	1,024<br>100.00

166 . tab cs_privacy_concerns cs_sex, col

Key
frequency<br>*column percentage*

Would you have any privacy concerns about being sent a letter directly by a heal	Sex<br>Male	Female	Other	Total
No concerns	429<br>85.46	436<br>84.82	4<br>100.00	869<br>85.20
A little concern (ple	47<br>9.36	56<br>10.89	0<br>0.00	103<br>10.10
Significant concerns	26<br>5.18	22<br>4.28	0<br>0.00	48<br>4.71
Total	502<br>100.00	514<br>100.00	4<br>100.00	1,020<br>100.00

167 . tab cs_privacy_concerns cs_area_type, col

Key
frequency<br>*column percentage*

Would you have any privacy concerns about being sent a letter directly by a heal	I live in an area that is Rural	Urban	Total
No concerns	240<br>91.25	614<br>82.75	854<br>84.98
A little concern (ple	16<br>6.08	87<br>11.73	103<br>10.25
Significant concerns	7<br>2.66	41<br>5.53	48<br>4.78
Total	263<br>100.00	742<br>100.00	1,005<br>100.00

168 . tab cs_privacy_concerns cs_state, col

Key
frequency<br>*column percentage*

Would you have any privacy concerns about being sent a letter directly by a heal	State of residence Australia	New South	Northern	Queenslan	South Aus	Tasmania	Victoria	Western A	T[illegible]
No concerns	20<br>90.91	277<br>85.23	2<br>100.00	172<br>85.57	68<br>83.95	20<br>100.00	216<br>85.04	78<br>82.11	[illegible]<br>8[illegible]
A little concern (ple	2<br>9.09	31<br>9.54	0<br>0.00	17<br>8.46	10<br>12.35	0<br>0.00	28<br>11.02	12<br>12.63	[illegible]<br>1[illegible]
Significant concerns	0<br>0.00	17<br>5.23	0<br>0.00	12<br>5.97	3<br>3.70	0<br>0.00	10<br>3.94	5<br>5.26	[illegible]<br>[illegible]
Total	22<br>100.00	325<br>100.00	2<br>100.00	201<br>100.00	81<br>100.00	20<br>100.00	254<br>100.00	95<br>100.00	1[illegible]<br>10[illegible]

169 . tab cs_privacy_concerns cs_age_range, col

Key
frequency<br>*column percentage*

Would you have any privacy concerns about being sent a letter directly by a heal	cs_age_range 18-24	25-40	41-60	61-80	>81	Total
No concerns	89<br>89.00	232<br>84.06	313<br>83.24	209<br>86.72	14<br>100.00	857<br>85.10
A little concern (ple	9<br>9.00	33<br>11.96	43<br>11.44	18<br>7.47	0<br>0.00	103<br>10.23
Significant concerns	2<br>2.00	11<br>3.99	20<br>5.32	14<br>5.81	0<br>0.00	47<br>4.67
Total	100<br>100.00	276<br>100.00	376<br>100.00	241<br>100.00	14<br>100.00	1,007<br>100.00

170 . tab cs_privacy_concerns cs_thoughts, col

Key
frequency   *column percentage*

Would you have any privacy concerns about being sent a letter directly by a heal	What are your thoughts about being told (by a health professional) about genetic I would w	I would w	I would n	Total
No concerns	**431**<br>**86.90**	**322**<br>**85.64**	**125**<br>**79.11**	**878**<br>**85.24**
A little concern (ple	**48**<br>**9.68**	**40**<br>**10.64**	**16**<br>**10.13**	**104**<br>**10.10**
Significant concerns	**17**<br>**3.43**	**14**<br>**3.72**	**17**<br>**10.76**	**48**<br>**4.66**
Total	**496**<br>**100.00**	**376**<br>**100.00**	**158**<br>**100.00**	**1,030**<br>**100.00**

171 . tab cs_privacy_concerns cs_non_priv_concerns, col

Key
frequency   *column percentage*

Would you have any privacy concerns about being sent a letter directly by a heal	Would you have any other (non-privacy related) concerns about being sent a lette No concer	A little	Significa	Total
No concerns	**855**<br>**90.38**	**22**<br>**38.60**	**1**<br>**3.70**	**878**<br>**85.24**
A little concern (ple	**73**<br>**7.72**	**29**<br>**50.88**	**2**<br>**7.41**	**104**<br>**10.10**
Significant concerns	**18**<br>**1.90**	**6**<br>**10.53**	**24**<br>**88.89**	**48**<br>**4.66**
Total	**946**<br>**100.00**	**57**<br>**100.00**	**27**<br>**100.00**	**1,030**<br>**100.00**

172 .
173 . tab cs_non_priv_concerns cs_age_range, col

Key
frequency   *column percentage*

Would you have any other (non-privacy related) concerns about being sent a lette	cs_age_range 18-24	25-40	41-60	61-80	>81	Total
No concerns	**95**<br>**95.00**	**250**<br>**90.58**	**344**<br>**91.49**	**222**<br>**92.12**	**14**<br>**100.00**	**925**<br>**91.86**
A little concern (ple	**5**<br>**5.00**	**19**<br>**6.88**	**19**<br>**5.05**	**12**<br>**4.98**	**0**<br>**0.00**	**55**<br>**5.46**
Significant concerns	**0**<br>**0.00**	**7**<br>**2.54**	**13**<br>**3.46**	**7**<br>**2.90**	**0**<br>**0.00**	**27**<br>**2.68**
Total	**100**<br>**100.00**	**276**<br>**100.00**	**376**<br>**100.00**	**241**<br>**100.00**	**14**<br>**100.00**	**1,007**<br>**100.00**

174 . tab cs_non_priv_concerns cs_thoughts, col

Key
frequency<br>*column percentage*

Would you have any other (non-privacy related) concerns about being sent a lette	What are your thoughts about being told (by a health professional) about genetic I would w	I would w	I would n	Total
No concerns	**459**<br>**92.54**	**346**<br>**92.02**	**141**<br>**89.24**	**946**<br>**91.84**
A little concern (ple	**25**<br>**5.04**	**23**<br>**6.12**	**9**<br>**5.70**	**57**<br>**5.53**
Significant concerns	**12**<br>**2.42**	**7**<br>**1.86**	**8**<br>**5.06**	**27**<br>**2.62**
Total	**496**<br>**100.00**	**376**<br>**100.00**	**158**<br>**100.00**	**1,030**<br>**100.00**

175 . tab cs_non_priv_concerns cs_edu, col

Key
frequency<br>*column percentage*

Would you have any other (non-privacy related) concerns about being sent a lette	Highest level of education attained Before ye	Year 12	TAFE/dipl	Undergrad	Post-grad	Total
No concerns	**127**<br>**94.07**	**179**<br>**96.24**	**304**<br>**92.97**	**221**<br>**89.47**	**109**<br>**84.50**	**940**<br>**91.80**
A little concern (ple	**6**<br>**4.44**	**5**<br>**2.69**	**17**<br>**5.20**	**15**<br>**6.07**	**14**<br>**10.85**	**57**<br>**5.57**
Significant concerns	**2**<br>**1.48**	**2**<br>**1.08**	**6**<br>**1.83**	**11**<br>**4.45**	**6**<br>**4.65**	**27**<br>**2.64**
Total	**135**<br>**100.00**	**186**<br>**100.00**	**327**<br>**100.00**	**247**<br>**100.00**	**129**<br>**100.00**	**1,024**<br>**100.00**

176 .
177 . tab cs_contact_ways_preference cs_age_range, col

Key
frequency<br>*column percentage*

Which of these ways of being contacted by a health professional would you prefer	18-24	25-40	cs_age_range<br>41-60	61-80	>81	Total
None	**4**<br>**4.00**	**11**<br>**3.99**	**16**<br>**4.26**	**10**<br>**4.15**	**0**<br>**0.00**	**41**<br>**4.07**
A phone call	**22**<br>**22.00**	**62**<br>**22.46**	**78**<br>**20.74**	**50**<br>**20.75**	**3**<br>**21.43**	**215**<br>**21.35**
A text message	**17**<br>**17.00**	**31**<br>**11.23**	**16**<br>**4.26**	**6**<br>**2.49**	**0**<br>**0.00**	**70**<br>**6.95**
A letter by post	**32**<br>**32.00**	**87**<br>**31.52**	**152**<br>**40.43**	**127**<br>**52.70**	**9**<br>**64.29**	**407**<br>**40.42**
A letter by email	**22**<br>**22.00**	**76**<br>**27.54**	**96**<br>**25.53**	**44**<br>**18.26**	**0**<br>**0.00**	**238**<br>**23.63**
No preference	**3**<br>**3.00**	**9**<br>**3.26**	**18**<br>**4.79**	**4**<br>**1.66**	**2**<br>**14.29**	**36**<br>**3.57**
Total	**100**<br>**100.00**	**276**<br>**100.00**	**376**<br>**100.00**	**241**<br>**100.00**	**14**<br>**100.00**	**1,007**<br>**100.00**

178 . tab cs_contact_ways_preference cs_area_type, col

Key
frequency<br>*column percentage*

Which of these ways of being contacted by a health professional would you prefer	I live in an area that is<br>Rural	 Urban	Total
None	**9**<br>**3.42**	**30**<br>**4.04**	**39**<br>**3.88**
A phone call	**65**<br>**24.71**	**154**<br>**20.75**	**219**<br>**21.79**
A text message	**22**<br>**8.37**	**48**<br>**6.47**	**70**<br>**6.97**
A letter by post	**110**<br>**41.83**	**297**<br>**40.03**	**407**<br>**40.50**
A letter by email	**45**<br>**17.11**	**190**<br>**25.61**	**235**<br>**23.38**
No preference	**12**<br>**4.56**	**23**<br>**3.10**	**35**<br>**3.48**
Total	**263**<br>**100.00**	**742**<br>**100.00**	**1,005**<br>**100.00**

179 . tab cs_contact_ways_preference cs_sex, col

Key
frequency<br>*column percentage*

Which of these ways of being contacted by a health professional would you prefer	Sex<br>Male	Female	Other	Total
None	**29**<br>**5.78**	**13**<br>**2.53**	**0**<br>**0.00**	**42**<br>**4.12**
A phone call	**107**<br>**21.31**	**111**<br>**21.60**	**0**<br>**0.00**	**218**<br>**21.37**
A text message	**34**<br>**6.77**	**37**<br>**7.20**	**1**<br>**25.00**	**72**<br>**7.06**
A letter by post	**198**<br>**39.44**	**213**<br>**41.44**	**1**<br>**25.00**	**412**<br>**40.39**
A letter by email	**112**<br>**22.31**	**125**<br>**24.32**	**2**<br>**50.00**	**239**<br>**23.43**
No preference	**22**<br>**4.38**	**15**<br>**2.92**	**0**<br>**0.00**	**37**<br>**3.63**
Total	**502**<br>**100.00**	**514**<br>**100.00**	**4**<br>**100.00**	**1,020**<br>**100.00**

180 .
181 . tab cs_fam_contact_preference cs_sex, col

Key
frequency<br>*column percentage*

If a health profession al contacted you in one of these ways, would you prefer th	Sex<br>Male	Female	Other	Total
No	**185**<br>**36.85**	**137**<br>**26.65**	**3**<br>**75.00**	**325**<br>**31.86**
Yes	**214**<br>**42.63**	**254**<br>**49.42**	**1**<br>**25.00**	**469**<br>**45.98**
Unsure	**103**<br>**20.52**	**123**<br>**23.93**	**0**<br>**0.00**	**226**<br>**22.16**
Total	**502**<br>**100.00**	**514**<br>**100.00**	**4**<br>**100.00**	**1,020**<br>**100.00**

182 . tab cs_fam_contact_preference cs_age_range,col

Key
frequency<br>*column percentage*

If a health professional contacted you in one of these ways, would you prefer th	cs_age_range 18-24	25-40	41-60	61-80	>81	Total
No	**30**<br>**30.00**	**86**<br>**31.16**	**108**<br>**28.72**	**93**<br>**38.59**	**5**<br>**35.71**	**322**<br>**31.98**
Yes	**52**<br>**52.00**	**124**<br>**44.93**	**166**<br>**44.15**	**112**<br>**46.47**	**8**<br>**57.14**	**462**<br>**45.88**
Unsure	**18**<br>**18.00**	**66**<br>**23.91**	**102**<br>**27.13**	**36**<br>**14.94**	**1**<br>**7.14**	**223**<br>**22.14**
Total	**100**<br>**100.00**	**276**<br>**100.00**	**376**<br>**100.00**	**241**<br>**100.00**	**14**<br>**100.00**	**1,007**<br>**100.00**

183 .
184 . tab cs_letter1_thoughts cs_letter2_thoughts, col

Key
frequency<br>*column percentage*

You may have noticed that the letter (Letter 1) did not say which genetic condit	This letter (Letter 2) is similar to Letter 1 but contains more information abou<br>I felt be	It felt o	Other (pl	Total
I would have felt bet	**662**<br>**85.42**	**83**<br>**35.93**	**4**<br>**16.67**	**749**<br>**72.72**
Including the genetic	**103**<br>**13.29**	**146**<br>**63.20**	**1**<br>**4.17**	**250**<br>**24.27**
Other (please elabora	**10**<br>**1.29**	**2**<br>**0.87**	**19**<br>**79.17**	**31**<br>**3.01**
Total	**775**<br>**100.00**	**231**<br>**100.00**	**24**<br>**100.00**	**1,030**<br>**100.00**

185 . tab cs_letter1_thoughts cs_letter_preference, col

Key
frequency<br>*column percentage*

You may have noticed that the letter (Letter 1) did not say which genetic condit	Having read both letters, which letter do you prefer? Letter 1	Letter 2	No prefer	Total
I would have felt bet	**114**<br>**52.53**	**568**<br>**82.20**	**67**<br>**54.92**	**749**<br>**72.72**
Including the genetic	**99**<br>**45.62**	**113**<br>**16.35**	**38**<br>**31.15**	**250**<br>**24.27**
Other (please elabora	**4**<br>**1.84**	**10**<br>**1.45**	**17**<br>**13.93**	**31**<br>**3.01**
Total	**217**<br>**100.00**	**691**<br>**100.00**	**122**<br>**100.00**	**1,030**<br>**100.00**

```
186 . tab cs_letter2_thoughts cs_letter_preference, col
```

Key
frequency<br>*column percentage*

This letter (Letter 2) is similar to Letter 1 but contains more information abou	Having read both letters, which letter do you prefer? Letter 1	Letter 2	No prefer	Total
I felt better informe	**99**<br>**45.62**	**613**<br>**88.71**	**63**<br>**51.64**	**775**<br>**75.24**
It felt overwhelming	**114**<br>**52.53**	**73**<br>**10.56**	**44**<br>**36.07**	**231**<br>**22.43**
Other (please elabora	**4**<br>**1.84**	**5**<br>**0.72**	**15**<br>**12.30**	**24**<br>**2.33**
Total	**217**<br>**100.00**	**691**<br>**100.00**	**122**<br>**100.00**	**1,030**<br>**100.00**

```
187 .
188 .
189 .
190 . *********************Graphs********************
191 .
192 . graph bar (count), over(cs_thoughts)

193 . graph export "graphs/bar_thoughts.png", as(png) name("Graph") replace
    file /Users/keri.finlay/Documents/Stata/graphs/bar_thoughts.png saved as PNG format

194 .
195 . graph bar (count) cs_letter1_thoughts cs_letter2_thoughts, over (cs_thoughts)

196 . graph export "graphs/bar_letter12thoughts.png", as(png) name("Graph") replace
    file /Users/keri.finlay/Documents/Stata/graphs/bar_letter12thoughts.png saved as PNG format

197 .
198 . generate cs_contact_pref_1 = 1 if cs_contact_ways == "1", after(cs_contact_ways)
    (909 missing values generated)
```

```
199 . replace cs_contact_pref_1 = 1 if cs_contact_ways == "1,2"
    (23 real changes made)

200 . replace cs_contact_pref_1 = 1 if cs_contact_ways == "1,3"
    (81 real changes made)

201 . replace cs_contact_pref_1 = 1 if cs_contact_ways == "1,4"
    (26 real changes made)

202 . replace cs_contact_pref_1 = 1 if cs_contact_ways == "1,2,3"
    (10 real changes made)

203 . replace cs_contact_pref_1 = 1 if cs_contact_ways == "1,3,4"
    (64 real changes made)

204 . replace cs_contact_pref_1 = 1 if cs_contact_ways == "1,2,4"
    (3 real changes made)

205 . replace cs_contact_pref_1 = 1 if cs_contact_ways =="1,2,3,4"
    (39 real changes made)

206 . generate cs_contact_pref_2= 2 if cs_contact_ways == "2", after(cs_contact_pref_1)
    (976 missing values generated)

207 . replace cs_contact_pref_2 = 2 if cs_contact_ways == "1,2"
    (23 real changes made)

208 . replace cs_contact_pref_2 = 2 if cs_contact_ways == "2,3"
    (5 real changes made)

209 . replace cs_contact_pref_2 = 2 if cs_contact_ways == "2,4"
    (8 real changes made)

210 . replace cs_contact_pref_2 = 2 if cs_contact_ways == "1,2,3"
    (10 real changes made)

211 . replace cs_contact_pref_2 = 2 if cs_contact_ways == "1,2,4"
    (3 real changes made)

212 . replace cs_contact_pref_2 = 2 if cs_contact_ways == "2,3,4"
    (16 real changes made)

213 . replace cs_contact_pref_2 = 2 if cs_contact_ways =="1,2,3,4"
    (39 real changes made)

214 . generate cs_contact_pref_3 = 3 if cs_contact_ways == "3", after(cs_contact_pref_2)
    (727 missing values generated)

215 . replace cs_contact_pref_3 = 3 if cs_contact_ways == "1,3"
    (81 real changes made)

216 . replace cs_contact_pref_3 = 3 if cs_contact_ways == "2,3"
    (5 real changes made)

217 . replace cs_contact_pref_3 = 3 if cs_contact_ways == "3,4"
    (121 real changes made)

218 . replace cs_contact_pref_3 = 3 if cs_contact_ways == "1,2,3"
    (10 real changes made)

219 . replace cs_contact_pref_3 = 3 if cs_contact_ways == "1,3,4"
    (64 real changes made)
```

```
220 . replace cs_contact_pref_3 = 3 if cs_contact_ways == "2,3,4"
    (16 real changes made)

221 . replace cs_contact_pref_3 = 3 if cs_contact_ways =="1,2,3,4"
    (39 real changes made)

222 . generate cs_contact_pref_4 = 4 if cs_contact_ways == "4", after(cs_contact_pref_3)
    (928 missing values generated)

223 . replace cs_contact_pref_4 = 4 if cs_contact_ways == "1,4"
    (26 real changes made)

224 . replace cs_contact_pref_4 = 4 if cs_contact_ways == "2,4"
    (8 real changes made)

225 . replace cs_contact_pref_4 = 4 if cs_contact_ways == "3,4"
    (121 real changes made)

226 . replace cs_contact_pref_4 = 4 if cs_contact_ways == "1,2,4"
    (3 real changes made)

227 . replace cs_contact_pref_4 = 4 if cs_contact_ways == "1,3,4"
    (64 real changes made)

228 . replace cs_contact_pref_4 = 4 if cs_contact_ways == "2,3,4"
    (16 real changes made)

229 . replace cs_contact_pref_4 = 4 if cs_contact_ways =="1,2,3,4"
    (39 real changes made)

230 .
231 . tab cs_contact_pref_1
```

cs_contact_ pref_1	Freq.	Percent	Cum.
1	367	100.00	100.00
Total	367	100.00	

```
232 . tab cs_contact_pref_2
```

cs_contact_ pref_2	Freq.	Percent	Cum.
2	158	100.00	100.00
Total	158	100.00	

```
233 . tab cs_contact_pref_3
```

cs_contact_ pref_3	Freq.	Percent	Cum.
3	639	100.00	100.00
Total	639	100.00	

```
234 . tab cs_contact_pref_4
```

cs_contact_ pref_4	Freq.	Percent	Cum.
4	379	100.00	100.00
Total	379	100.00	

```
235 .
236 . graph bar (count) cs_contact_pref_1 cs_contact_pref_2 cs_contact_pref_3 cs_contact_pref_4

237 . graph export "graphs/bar_contact_pref.png", as(png) name("Graph") replace
    file /Users/keri.finlay/Documents/Stata/graphs/bar_contact_pref.png saved as PNG format

238 .
239 . *****************signifance tests***************
240 . tab cs_edu cs_privacy_concerns, chi
```

Highest level of education attained	Would you have any privacy concerns about being sent a letter directly by a heal: No concer	A little	Significa	Total
Before year 12	**122**	**8**	**5**	**135**
Year 12	**163**	**19**	**4**	**186**
TAFE/diploma	**289**	**29**	**9**	**327**
Undergraduate degree	**197**	**32**	**18**	**247**
Post-graduate degree	**101**	**16**	**12**	**129**
Total	**872**	**104**	**48**	**1,024**

Pearson chi2(**8**) = **22.8508** Pr = **0.004**

```
241 . tab cs_edu cs_privacy_concerns, exp
```

Key
frequency
expected frequency

Highest level of education attained	Would you have any privacy concerns about being sent a letter directly by a heal: No concer	A little	Significa	Total
Before year 12	**122**<br>**115.0**	**8**<br>**13.7**	**5**<br>**6.3**	**135**<br>**135.0**
Year 12	**163**<br>**158.4**	**19**<br>**18.9**	**4**<br>**8.7**	**186**<br>**186.0**
TAFE/diploma	**289**<br>**278.5**	**29**<br>**33.2**	**9**<br>**15.3**	**327**<br>**327.0**
Undergraduate degree	**197**<br>**210.3**	**32**<br>**25.1**	**18**<br>**11.6**	**247**<br>**247.0**
Post-graduate degree	**101**<br>**109.9**	**16**<br>**13.1**	**12**<br>**6.0**	**129**<br>**129.0**
Total	**872**<br>**872.0**	**104**<br>**104.0**	**48**<br>**48.0**	**1,024**<br>**1,024.0**

```
242 . tab cs_edu cs_non_priv_concerns, chi
```

Highest level of education attained	Would you have any other (non-privacy related) concerns about being sent a lette: No concer	A little	Significa	Total
Before year 12	**127**	**6**	**2**	**135**
Year 12	**179**	**5**	**2**	**186**
TAFE/diploma	**304**	**17**	**6**	**327**
Undergraduate degree	**221**	**15**	**11**	**247**
Post-graduate degree	**109**	**14**	**6**	**129**
Total	**940**	**57**	**27**	**1,024**

Pearson chi2(**8**) = **19.4392** Pr = **0.013**

243 . tab cs_edu cs_non_priv_concerns, exp

Key
frequency<br>*expected frequency*

Highest level of education attained	Would you have any other (non-privacy related) concerns about being sent a lette<br>No concer	A little	Significa	Total
Before year 12	**127**<br>**123.9**	**6**<br>**7.5**	**2**<br>**3.6**	**135**<br>**135.0**
Year 12	**179**<br>**170.7**	**5**<br>**10.4**	**2**<br>**4.9**	**186**<br>**186.0**
TAFE/diploma	**304**<br>**300.2**	**17**<br>**18.2**	**6**<br>**8.6**	**327**<br>**327.0**
Undergraduate degree	**221**<br>**226.7**	**15**<br>**13.7**	**11**<br>**6.5**	**247**<br>**247.0**
Post-graduate degree	**109**<br>**118.4**	**14**<br>**7.2**	**6**<br>**3.4**	**129**<br>**129.0**
Total	**940**<br>**940.0**	**57**<br>**57.0**	**27**<br>**27.0**	**1,024**<br>**1,024.0**

244 . tab cs_edu cs_letter_preference, chi

Highest level of education attained	Having read both letters, which letter do you prefer?<br>Letter 1	Letter 2	No prefer	Total
Before year 12	**28**	**85**	**22**	**135**
Year 12	**36**	**129**	**21**	**186**
TAFE/diploma	**74**	**214**	**39**	**327**
Undergraduate degree	**52**	**175**	**20**	**247**
Post-graduate degree	**26**	**85**	**18**	**129**
Total	**216**	**688**	**120**	**1,024**

Pearson chi2(**8**) = **7.6260** Pr = **0.471**

245 . tab cs_edu cs_letter2_difficulty, chi

Highest level of education attained	How easy was Letter 2 to understand?<br>Very easy	Easy enou	A bit dif	Very diff	Total
Before year 12	**56**	**68**	**11**	**0**	**135**
Year 12	**76**	**86**	**22**	**2**	**186**
TAFE/diploma	**144**	**153**	**26**	**4**	**327**
Undergraduate degree	**109**	**115**	**21**	**2**	**247**
Post-graduate degree	**66**	**49**	**10**	**4**	**129**
Total	**451**	**471**	**90**	**12**	**1,024**

Pearson chi2(**12**) = **12.9759** Pr = **0.371**

246 . tab cs_edu cs_letter1_difficulty, chi

Highest level of education attained	How easy was Letter 1 to understand? Very easy	Easy enou	A bit dif	Very diff	Total
Before year 12	50	68	17	0	135
Year 12	70	97	14	5	186
TAFE/diploma	142	158	21	6	327
Undergraduate degree	107	118	21	1	247
Post-graduate degree	55	55	15	4	129
Total	424	496	88	16	1,024

Pearson chi2(12) = 17.1848 Pr = 0.143

247 . tab cs_age_range cs_letter_preference, chi

cs_age_range	Having read both letters, which letter do you prefer? Letter 1	Letter 2	No prefer	Total
18-24	26	63	11	100
25-40	70	181	25	276
41-60	80	245	51	376
61-80	32	178	31	241
>81	1	11	2	14
Total	209	678	120	1,007

Pearson chi2(8) = 17.4612 Pr = 0.026

248 . tab cs_area_type cs_thoughts, chi

I live in an area that is	What are your thoughts about being told (by a health professional) about genetic I would w	I would w	I would n	Total
Rural	117	102	44	263
Urban	365	269	108	742
Total	482	371	152	1,005

Pearson chi2(2) = 1.8400 Pr = 0.399

249 . tab cs_fam_contact_preference cs_sex, chi

If a health professional contacted you in one of these ways, would you prefer th	Sex Male	Female	Other	Total
No	185	137	3	325
Yes	214	254	1	469
Unsure	103	123	0	226
Total	502	514	4	1,020

Pearson chi2(4) = 15.7888 Pr = 0.003

```
250 .
    end of do-file

251 . log close
          name:  <unnamed>
           log:  /Users/keri.finlay/Documents/Stata/logfile_survey.smcl
      log type:  smcl
     closed on:  15 Sep 2022, 13:46:36
```
